# AlGaAs Nonlinear Integrated Photonics

**DOI:** 10.3390/mi13070991

**Published:** 2022-06-24

**Authors:** Ehsan Mobini, Daniel H. G. Espinosa, Kaustubh Vyas, Ksenia Dolgaleva

**Affiliations:** 1 Department of Physics, University of Ottawa, Ottawa, ON K1N 6N5, Canada; eh.mobini@gmail.com; 2 School of Electrical Engineering and Computer Science, University of Ottawa, Ottawa, ON K1N 6N5, Canada; daniel.espinosa@uottawa.ca (D.H.G.E.); kvyas027@uottawa.ca (K.V.)

**Keywords:** nonlinear optics, integrated photonics, AlGaAs platform, four-wave mixing

## Abstract

Practical applications implementing integrated photonic circuits can benefit from nonlinear optical functionalities such as wavelength conversion, all-optical signal processing, and frequency-comb generation, among others. Numerous nonlinear waveguide platforms have been explored for these roles; the group of materials capable of combining both passive and active functionalities monolithically on the same chip is III–V semiconductors. AlGaAs is the most studied III–V nonlinear waveguide platform to date; it exhibits both second- and third-order optical nonlinearity and can be used for a wide range of integrated nonlinear photonic devices. In this review, we conduct an extensive overview of various AlGaAs nonlinear waveguide platforms and geometries, their nonlinear optical performances, as well as the measured values and wavelength dependencies of their effective nonlinear coefficients. Furthermore, we highlight the state-of-the-art achievements in the field, among which are efficient tunable wavelength converters, on-chip frequency-comb generation, and ultra-broadband on-chip supercontinuum generation. Moreover, we overview the applications in development where AlGaAs nonlinear functional devices aspire to be the game-changers. Among such applications, there is all-optical signal processing in optical communication networks and integrated quantum photonic circuits.

## 1. Introduction

The impact of photonics in the 21st century is as significant as the influence of the “Electronics Age” of the past 60 years. Photonic integration offers scalability of complex optical setups; unprecedented robustness, energy, and cost efficiency; finding niches in medical diagnostics; chemical and environmental sensing; spectroscopy; optical communication networks; quantum information; and many other applications. One of the benefits of photonic integration is the realization of compact highly efficient low-power on-chip nonlinear optical devices that can augment the performance of the existing commercial photonic integration circuits.

Let us consider, as one particular example, optical communication networks application. The present role of large-scale photonic integration is to replace a large number of bulk components at the optical-to-electrical and back-to-optical (OEO) converters at the network nodes with compact optical transceiver chips. Along these lines, for example, Infenera has recently demonstrated a coherent transmitter with over 4.9 Tb/s of capacity [1]. However, modern communication systems rely on electronic signal processing that has fundamental limitations associated with the bandwidth, power dissipation, and switching speed. The introduction of emerging technologies including autonomous vehicles and Smart Cities will pose additional challenges on communication networks. The current needs and future challenges faced by industries which are based on or rely on information and communication technologies can be addressed through an all-optical signal processing approach [2,3,4,5,6] with integrated photonic circuits.

All-optical signal processing operations rely predominantly on all-optical wavelength conversion, which can be implemented using nonlinear optical effects to modify the spectrum of the optical signal. The most significant of these effects for wavelength conversion, four-wave mixing (FWM), entails nonlinear optical interaction of two or more spectral components (light frequencies) within a nonlinear optical medium, which produces a new frequency component as an outcome of this interaction. The FWM-based wavelength conversion offers unprecedented transparency with respect to the signal modulation format [7], which is especially important for modern communication networks relying on complex modulation schemes. Although there are many materials that can be used to produce this nonlinear interaction [2,3,4,5,6], a III–V semiconductor compound aluminum gallium arsenide (AlGaA) has consistently been shown to demonstrate exceptional qualities for all-optical signal processing at the Telecom C-band (frequency range centered around 1550 nm, the fiber optics wavelength). Coupled with the fact that it can produce the strongest nonlinear optical interaction for wavelength conversion [8,9,10,11], it exhibits low linear and nonlinear propagation losses in the Telecom spectral range (1400–1600 nm) [9,12,13], which is highly desirable in a commercial context. Furthermore, AlGaAs exhibits strong electro-optic effect, beneficial for fast optical signal routing, and a large thermo-optic coefficient (two times larger than that of Si), enabling efficient thermal tuning. All these qualities puts AlGaAs in a unique position for practical realization of all-optical functions. This review is focused on the progress and achievements in the field of AlGaAs nonlinear photonics.

The first experiment on frequency conversion in a planar waveguide was reported by D. B. Anderson and J. T. Boyd in 1971 where the authors used a GaAs thin film as the nonlinear medium [14]. This study was followed by other nonlinear optical experiments performed in GaAs and AlGaAs waveguides [13,15,16,17,18,19,20,21,22,23,24,25,26,27,28,29,30,31,32,33,34,35,36,37,38,39,40,41,42,43,44,45,46,47,48,49,50,51,52], as well as in InP-based nonlinear waveguide platforms [53,54,55,56,57,58]. In such a way, III–V semiconductors were the first waveguide platforms explored in nonlinear optics.

Since the early nonlinear waveguide experiments, there has been a continuing quest for new nonlinear waveguide materials and waveguide platforms with optimized nonlinear optical performances. Silicon, widely recognized for integrating electronic and optical devices on a single chip, entered the nonlinear optics scene in the early 2000s. The first silicon-based nonlinear waveguide platform was silicon-on-insulator (SOI) [59,60]. In order to mitigate some limitations associated with SOI, such as its strong two-photon (2PA) and free-carrier (FCA) absorption in the wavelength range below 2.2 μm [61,62], other silicon-based nonlinear platforms such as Si${}_{3}$N${}_{4}$ [63,64,65,66], silicon-rich nitride [67,68,69,70], and amorphous silicon [71,72] have been developed and used to demonstrate nonlinear optical performances superior to that of SOI. Among other powerful photonic integration platforms exhibiting promising nonlinear optical properties are LiNbO${}_{3}$ with its strong second-order nonlinear optical response and electrooptic effect [73,74,75,76], chalcogenide glasses (ChG) with their strong third-order nonlinear optical responses [77,78,79,80,81], and Hydex glass with its ultra-low propagation losses and CMOS compatibility [82,83,84,85], to name a few. Among all these nonlinear waveguide materials, III–V semiconductors stand out due to their natural suitability for monolithic integration of passive and active nonlinear optical devices. They can accommodate passive waveguides for light steering and nonlinear manipulation, laser sources, modulators, and detectors monolithically on the same chip. This fact served as a driver stimulating further development of nonlinear optical devices based on III–V semiconductors and addressing challenges associated with loss mitigation and nonlinearity enhancement in these platforms.

Among the III–V integrated nonlinear photonic platforms considered to date are GaAs and its AlGaAs derivative, InP and InGaAsP [86], III-nitrides AlN [87,88,89] and GaN [87,90,91,92], as well as GaP [93] and its ternary derivative InGaP [94,95,96]. In this review, we focus on the most explored in nonlinear optics III–V semiconductor Al${}_{x}$Ga${}_{1-x}$As, which can be epitaxially grown on a GaAs substrate with a minimal lattice mismatch for the entire composition range 0≤x≤1. Further in the paper, where we speak about both GaAs and AlGaAs, we name them (Al)GaAs. We consolidate the information available on nonlinear optical coefficients of AlGaAs compounds and provide an overview of various (Al)GaAs waveguide platforms and geometries and their respective nonlinear optical performances. One of the most recently invented (Al)GaAs nonlinear waveguide platforms is (Al)GaAs-on-insulator [(Al)GaAs-OI], named after its resemblance with SOI. We dedicate special attention to (Al)GaAs-OI, describing the best-performing nonlinear photonic devices based on this platform. We also present the state-of-the-art of (Al)GaAs nonlinear integrated photonics and its applications, such as fully integrated wavelength converters, all-optical signal processing, and integrated quantum photonic circuits. Furthermore, we highlight the existing challenges in the development of practical AlGaAs nonlinear photonic circuits, namely: the technological compatibility of various active and passive integrated photonic components with nonlinear waveguide platforms and other areas of development. What lies outside the scope of the paper is the detailed comparison of (Al)GaAs waveguide platforms with bulk nonlinear optical materials and hybrid waveguide structures, as well as the discussion of the compatibility of (Al)GaAs with two-dimensional structures such as graphene. While there are a few theoretical works dedicated to the latter topic [97,98], there are no associated experimental developments in (Al)GaAs photonics to date.

The rest of the paper is organized as follows. In Section 2, we present an overview of the optical properties of (Al)GaAs and their comparison to those of other common nonlinear waveguide platforms. In Section 3, we describe various (Al)GaAs waveguide platforms and geometries. There, we also define some parameters used to evaluate the nonlinear optical performances of waveguides. Section 4 is dedicated to the nanofabrication approaches used in (Al)GaAs waveguide optics. Section 5 focuses on the second-order ($\chi^{\left(2\right)}$-related) nonlinear optical phenomena realized in (Al)GaAs waveguides. In Section 6, we cover nonlinear optical phenomena associated with the third-order nonlinear susceptibility $\chi^{\left(3\right)}$, and the $\chi^{\left(5\right)}$-related nonlinear absorption. In both Section 5 and Section 6, we define the main nonlinear optical coefficients, review all the relevant information on (Al)GaAs nonlinear optical devices available to date, and present the measured nonlinear coefficient values in tables that can come in handy. We also provide wavelength dependences of some nonlinear optical parameters. Further, we highlight the studies in which the highest nonlinear conversion efficiencies have been reported and summarize them in the corresponding tables. Section 7 presents various applications of AlGaAs nonlinear photonic devices and their state-of-the-art. We also discuss challenges associated with developing marketable AlGaAs nonlinear integrated circuits and how these challenges are addressed in the most recent research. Finally, Section 8 is dedicated to concluding remarks. In Appendix A, we give a survey of the existing theories capable of describing wavelength and material composition dependences of some nonlinear optical parameters of III–V semiconductors.

## 2. Optical Properties of AlGaAs

Aluminum gallium arsenide ${\mathrm{Al}}_{x}{\mathrm{Ga}}_{1-x}\mathrm{As}$, where the fractions of Al and Ga are interchangeable, can be epitaxially grown for the entire composition range 0≤x≤1. The lattice constants of the constituting binary compounds GaAs and AlAs are very close, 5.65 Å and 5.66 Å, respectively, and the lattice matching is maintained for all the ${\mathrm{Al}}_{x}{\mathrm{Ga}}_{1-x}\mathrm{As}$ compositions. This allows for defect-free growth of AlGaAs heterostructures with an arbitrary number of layers with different Al concentrations.

AlGaAs exhibits a wide transparency window from near- to mid-infrared (MIR) (0.9–17 μm [99,100]). Its bandgap energy can be modified by altering the aluminium concentration. The direct bandgap is maintained for the Al concentration x<0.45, and its value can be determined from $E_{g}\left(x\right)=1.422+1.2475x$, where all the numerical coefficients have the units of electron volt (eV) [101].

The refractive index of ${\mathrm{Al}}_{x}{\mathrm{Ga}}_{1-x}\mathrm{As}$ is high; it ranges between 2.9 (for x=1) and 3.4 (for x=0) at the telecom C-band [99] and serves as a variable parameter in waveguide design. Ref. [99] presents a detailed method with the set of equations and coefficients to determine the refractive index of ${\mathrm{Al}}_{x}{\mathrm{Ga}}_{1-x}\mathrm{As}$ as a function of the composition, wavelength, and temperature. Thanks to its large Kerr coefficient (around $n_{2}=1.5\times 10^{-13}\;{\mathrm{cm}}^{2}/W$) [13] and second-order nonlinear susceptibility $\chi^{\left(2\right)}$ over 200 pm/V [102,103], AlGaAs has been termed “the silicon of nonlinear optics” [28]. To date, second- and third-order nonlinear optical phenomena associated with the corresponding nonlinear optical susceptibilities $\chi^{\left(2\right)}$ and $\chi^{\left(3\right)}$, such as second-harmonic generation (SHG) [10,11,14,15,16,30,37,41,43,44,45,46,100,104,105,106,107,108,109,110,111,112,113,114,115,116,117,118,119,120,121,122], sum-frequency generation (SFG) [17,48,123,124], difference-frequency generation (DFG) [38,42,47,124,125,126,127], spontaneous parametric down-conversion (SPDC) [128,129,130,131], four-wave mixing (FWM) [7,9,20,132,133,134,135,136,137,138,139,140,141,142,143,144], 2PA [13,19,23,114,145,146,147,148], self-phase modulation (SPM) [23,32,114,133,134,146,147,149,150], cross-phase modulation (XPM) [32,133,146,149], spontaneous four-wave mixing (SFWM) [151,152], spatial soliton formation [49], stimulated Raman scattering [153], supercontinuum generation (SCG) [154,155], Kerr frequency microcomb [8,156], and the $\chi^{\left(5\right)}$-related three-photon absorption (3PA) [13,19,27,134] have been experimentally investigated in AlGaAs waveguide platforms.

In Table 1, we present a comparison between various nonlinear waveguide materials. The table displays their optical coefficients, including the linear refractive index $n_{0}$, the largest tensor component of $\chi^{\left(2\right)}$ (where applicable), Kerr coefficient $n_{2}$, two-photon absorption coefficient $\alpha_{2}$, and the lowest achieved linear propagation loss coefficient in a waveguide $L_{\min}$. The materials exhibiting natural $\chi^{\left(2\right)}$ are also marked with an asterisk. All the parameters are reported at the wavelength 1550±5 nm, unless stated otherwise. The bottom line of the table reports $n_{2}$ of silica glass as a benchmark. The desirable qualities for a nonlinear waveguide platform are high refractive index and Kerr coefficient, and low 2PA and propagation loss at the wavelength of interest. Additionally, the ability to demonstrate second-order nonlinear interactions is of special value for many applications. We further compare the materials listed in the table in terms of these performance parameters and discuss additional considerations contributing to the overall suitability for nonlinear photonic circuits.

A practical nonlinear waveguide platform should exhibit a high refractive index for compact waveguides. Furthermore, adjustable $n_{0}$ can serve as an additional design parameter in waveguide platforms. Al${}_{x}$Ga${}_{1-x}$As with $n_{0}>3$ and the ability to adjust its value by changing *x* satisfies these requirements. Other materials that meet these requirements include ternary and quaternary III–V semiconductors In${}_{x}$Ga${}_{1-x}$As and In${}_{x}$Ga${}_{1-x}$As${}_{y}$P${}_{1-y}$. Silicon exhibits a high refractive index; however, there is no means for its adjustment.

All the materials listed in the table (except for silica glass) exhibit sufficiently high values of $n_{2}$. Out of these materials, ${\mathrm{Al}}_{0.18}{\mathrm{Ga}}_{0.82}\mathrm{As}$ and GaAs stand out, having the highest values of $n_{2}$. GaAs, however, is not suitable for nonlinear optical experiments at 1550 nm due to its relatively high $\alpha_{2}$ at that wavelength range. In contrast, the particular material composition ${\mathrm{Al}}_{0.18}{\mathrm{Ga}}_{0.82}\mathrm{As}$ of AlGaAs is often considered in nonlinear waveguide experiments at 1550 nm because the corresponding bandgap energy is blue-shifted such that $\alpha_{2}$ is very small. In such a way, ${\mathrm{Al}}_{0.18}{\mathrm{Ga}}_{0.82}\mathrm{As}$ represents a great alternative to GaAs binary in the telecom C-band, exhibiting almost as high $n_{2}$ at the expense of a very small $\alpha_{2}$. Some materials such as, e.g., Hydex glass, exhibit negligible 2PA at 1550 nm, not detectable in nonlinear experiments (see, e.g., [82]). However, their values of $n_{2}$ are 1–2 orders of magnitude smaller than those of AlGaAs and GaAs. Looking at the table, we can conclude that most materials exhibit a negligible or very small 2PA at 1550 nm, except for Si, GaAs, and InGaAsP. Therefore, the latter three compounds are not the best choice for nonlinear photonic devices operating in the telecom C-band.

Other III–V semiconductors used in nonlinear photonics also exhibit moderate to high values of $n_{2}$ ranging between $2.3\times 10^{-15}$ cm${}^{2}$/W for AlN and $1\times 10^{-13}$ cm${}^{2}$/W for InGaAsP. AlN, GaN, GaP, and InGaP are wide-bandgap semiconductors with transparency windows partially or fully covering the visible range. They represent a special interest in nonlinear photonics for their potential to be used in the visible spectral range. These material platforms are still in development for nonlinear photonic applications; more research is necessary to fully understand their potential for practical implementation.

The propagation loss has been a bottleneck factor in waveguide optics for a while, especially for AlGaAs heterostructure waveguides where the propagation loss coefficients reached 10 dB/mm in ultracompact devices [137], and 1 dB/cm in weakly-guiding strip-loaded waveguides [134]. Thanks to recent advances in fabrication process development and new fabrication approaches [9,12,117,120,142,176,177], it has become possible to minimize the propagation loss in AlGaAs waveguides. The newest waveguide platforms such as GaAs- and AlGaAs-on-insulator [(Al)GaAs-OI] [7,8,10,11,12,118,119,139,140,141,142,152,156,177], suspended (Al)GaAs [117,120,121,122] waveguides, can exhibit ultralow propagation loss coefficients ≪1 dB/cm, comparable to those demonstrated by SOI waveguides.

Another benefit that AlGaAs and other III–V semiconductors offer for nonlinear photonics is their suitability for even-order nonlinear optical interactions. Only noncentrosymmetric materials can naturally exhibit second-order nonlinear optical interactions. Most III–V semiconductors have Wurtzite or Zincblende crystalline structures lacking the center of inversion symmetry, which makes them efficient $\chi^{\left(2\right)}$ materials. GaAs and AlGaAs hold the values of $\chi^{\left(2\right)}$ among the highest of all III–V semiconductors. Another nonlinear waveguide platform exhibiting natural $\chi^{\left(2\right)}$ is LiNbO${}_{3}$. Furthermore, recent achievements in straining silicon enabled the observation of $\chi^{\left(2\right)}$ interactions in this material [168,175,178,179] with the value of $\chi^{\left(2\right)}$ comparable to that of LiNbO${}_{3}$. However, these $\chi^{\left(2\right)}$ values are at least five times smaller compared to that of GaAs [74,161].

Last but not least, most III–V semiconductors are direct-bandgap and are capable of emitting light, unlike the rest of the materials in Table 1. Combined with the ability to vary the material compositions and the bandgap energies in ternary and quaternary III–V semiconductors, their light-emitting capability is vital for monolithic integration. The epitaxial arrangement of a III–V semiconductor structure can be prepared in a way that the light sources, passive optical components and detectors all co-exist on the same material platform (see an example with InP [180,181]). These facts indicate that AlGaAs is a promising material for monolithically integrated photonic circuits with nonlinear functionalities.

It is important to remark before concluding this section that organic polymers represent another large group of waveguide materials, including a variety of representatives [182,183,184,185,186,187]. They can exhibit high values of $n_{2}$ ranging between $10^{-14}$ and $10^{-12}$ cm${}^{2}$/W, measured in the visible and near-IR wavelength ranges. Some of these materials with non-centrosymmetric molecules feature second-order nonlinear susceptibilities [187]. However, their relatively low refractive indices with typical values < 2 set the limit on the compactness of the waveguide structures and dispersion engineering.

More recently, the focus in polymer waveguide optics has been shifted towards hybrid silicon-organic waveguide structures [188,189] where silicon slot waveguides provide light confinement while the polymer materials serve as claddings. Other variations of hybrid waveguides with polymer claddings include, for example, LiNbO${}_{3}$ as an electrooptic material [190]. This configuration, however, has design restrictions and does not address the need for integrated light sources. Furthermore, hybrid waveguide structures are a separate topic lying outside the scope of this article. They do, however, represent a promising solution towards all-on-chip photonic circuits. Recent advances in hybrid integration of III–V semiconductors with silicon and silicon nitride rely on epitaxial approaches and demonstrate promising results. For more in-depth coverage of this topic, we refer the reader to the recent review articles [191,192].

## 3. AlGaAs Waveguide Platforms

**Figure 1:**
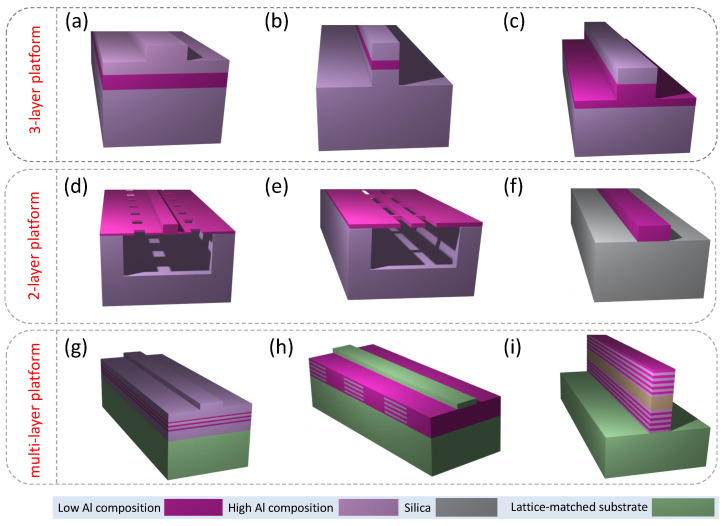
Schematics of AlGaAs waveguide platforms and geometries. (**a**–**c**) Three-layer platform waveguide geometries: strip-loaded, nanowire, and half-core waveguides, respectively. (**d**–**f**) Two-layer platforms including suspended nanorib, suspended nanowire, and AlGaAs-on-insulator waveguides, respectively. (**g**–**i**) Multi-layer platform including multi-quantum-well waveguide, modulated-$\chi^{2}$ waveguide, and Bragg-reflector waveguide, respectively. The waveguides shown in (**h**,**i**) are typically designed for phase matching of the $\chi^{\left(2\right)}$ processes.

AlGaAs platforms used in nonlinear integrated photonics can be divided into three-layer, two-layer, and multi-layer platforms, depending on the number of epitaxially grown layers with different material compositions (see Figure 1). The three-layer platforms are traditionally in the basis of strip-loaded [13,23,28,31,32,33,34,49,133,134,144], nanowire [9,135,136,137,138,144,149,151], and half-core [144] waveguide geometries (Figure 1a–c, respectively), mostly used for Kerr-type nonlinear devices. Modal-phase-matched SHG in nanowire waveguides has also been demonstrated [115].

Suspended nanorib [120,121], suspended nanowire [117,121,122], AlGaAs-OI [7,8,10,11,12,109,118,119,139,140,141,142,152,156,177] (Figure 1d–f, respectively), and AlGaAs ridge or rib waveguides [17,19] (they match the schematic in Figure 1) are examples of the waveguide geometries based on the two-layer platforms. They have been used for a variety of $\chi^{\left(2\right)}$-based [10,11,12,17,109,117,118,119,120] and $\chi^{\left(3\right)}$-based [7,8,19,139,140,141,142,152,156] nonlinear optical experiments. Multi-quantum-well and multilayer [19,37,41,42,43,44,46,47,100,105,107,124,128,146,150], modulated-$\chi^{2}$ [30,38,43,44,45,48,106,110,112,113,126,127], and Bragg-reflector [104,108,111,114,123,125,128,193] waveguides represent multi-layer waveguide platforms (Figure 1g–i, respectively). Many of these platforms are developed specifically for phase matching (PM) of the second-order nonlinear optical interactions.

In the following subsection, we discuss some nonlinear waveguide parameters that will help us to understand relative merit of various waveguide designs presented in Figure 1.

### 3.1. Nonlinear Parameters of a Waveguide

Here, we present and discuss two important nonlinear parameters relevant in evaluating the nonlinear optical performance of a waveguide. The first parameter is the effective mode area $A_{\mathrm{eff}}$ [194], given by the expression

(1) $$A_{\mathrm{eff}}=\frac{{\left(\;\int \int \;{\left|E\right|}^{2}\;dx\;dy\right)}^{2}}{\;\int \int \;{\left|E\right|}^{4}\;dx\;dy},$$

where E represents the electric field inside the waveguide, and the integration is performed over the entire cross-sectional plane xy. It is worthwhile mentioning here that the effective mode area is defined differently for different orders of the optical nonlinearity [13]. Equation (1) represents an effective mode area used in analyzing the third-order nonlinear optical processes. The degree of light confinement in a waveguide is related to the efficiency of the nonlinear optical interactions, as one can achieve different levels of intensity at the same level of the incident optical power. That is why the nonlinear optical waveguides with smaller $A_{\mathrm{eff}}$ tend to exhibit more efficient nonlinear optical interactions.

The second important parameter of a waveguide depending on its geometry and material characteristics is the nonlinear coefficient, defined as

(2) $$\gamma =\frac{2\pi n_{2}}{\lambda_{0}A_{\mathrm{eff}}},$$

where $\lambda_{0}$ is the wavelength of light in free space. As can be seen from Equation (2), the strength of the nonlinear optical interactions inside the waveguide depends on the employed Kerr medium and the geometrical design. The Kerr coefficient for AlGaAs, as mentioned earlier, is large compared to many other nonlinear materials, which results in larger γ coefficients realized in AlGaAs waveguides. Table 2 summarizes the measured values of the nonlinear coefficient of various AlGaAs waveguide platforms and geometries, described further.

Below we describe all the waveguide geometries shown in Figure 1 in the context of their overall nonlinear optical performances in more detail.

### 3.2. Strip-Loaded Waveguides

Strip-loaded ${\mathrm{Al}}_{x}{\mathrm{Ga}}_{1-x}\mathrm{As}$ waveguides [see Figure 1a] are composed of three epitaxial layers with different Al concentration [134]. The layer with the lowest Al concentration serves as the guiding layer as it has the highest refractive index. The lower-index (higher-Al) layers serve as claddings, providing light confinement in the vertical dimension. Lateral confinement is achieved by a ridge lithographically defined and partially etched in the upper cladding of the waveguide. Strip-loaded waveguide structure was one of the earliest waveguide structures with 2D light confinement explored in nonlinear optics. Strip-loaded waveguides have relatively low propagation loss coefficients (<1 dB/cm) and less critical requirements for the fabrication process [13,28]. They represent a weakly guiding waveguide structure with the refractive index contrast between the core and claddings, usually falling in the range Δn=0.03 to 0.2. This type of waveguide does not permit dispersion engineering [134] due to its low refractive index contrast and relatively large waveguide dimensions (typically >1 μm). These characteristics also result in relatively large effective mode areas ranging from 1.5 [144] to 12 [13] μm${}^{2}$ with the corresponding modest values of the nonlinear coefficient γ not higher than 50 m${}^{-1}$W${}^{-1}$ [144].

### 3.3. Nanowires

To overcome some of the shortcomings of strip-loaded waveguides, a different waveguide geometry known as nanowire [See Figure 1b], in which the guiding layer is etched through and the corresponding waveguide dimensions can be ≪1μm, was proposed by Meier et al. [195]. The corresponding effective mode area in such waveguides is reduced down to <0.5 μm${}^{2}$ [137], and the corresponding nonlinear coefficient can reach the value 250 m${}^{-1}$W${}^{-1}$ and higher [144]. This design features a sufficiently strong waveguide dispersion such that it can compensate and even overcompensate the material dispersion [137,195]. However, because the guiding layer where the light is confined is etched through, and the sidewalls are exposed to a very sharp refractive index contrast with the air, nanowires can suffer from very high propagation loss coefficients (10 dB/mm) due to the scattering off sidewall imperfections formed in the process of dry etching [115,137]. Nevertheless, recent improvements in waveguide fabrication process, such as sidewall passivation and plasma-assisted photoresist reflow [9,176], allowed one to bring the propagation loss coefficient down to an impressively low level of 0.56 dB/cm.

### 3.4. Half-Core Waveguides

The waveguide geometry that combines some of the benefits of strip-loaded and nanowire geometries, such as reduced propagation loss and a small effective mode area, can be realized by etching the waveguide core half-way [144]. This waveguide geometry is known as half-core (see Figure 1c). The effective mode area in such waveguides can be made <1 μm${}^{2}$ with the corresponding values of γ>100 m${}^{-1}$W${}^{-1}$ [144].

We remark here that all the geometries presented in Figure 1a–c are sometimes referred to as ridge waveguides in the literature. Strictly speaking, a ridge waveguide is a waveguide matching the structure represented in Figure 1f. Due to the difference in the nonlinear optical performances of the three geometries shown in Figure 1a–c, it is instructive to discriminate between the three of them and to differentiate them from the simpler ridge waveguide geometry of Figure 1f [17,19].

### 3.5. AlGaAs-OI

Three-layer heterostructures are the most accessible platform to work with from the fabrication standpoint. They require a simple single-step epitaxial growth and a single-step lithography/etching process. However, the difficulty associated with dispersion engineering, moderate refractive index contrasts in the vertical dimension, and lack of versatility of three-layer heterostructures persuaded researchers to develop AlGaAs waveguides on a non-native substrate, namely silica [177] and sapphire [142]. This approach provides a refractive index contrast Δn=1.9 at the telecom wavelength and allows one to achieve a very small effective mode area < 0.2 μm${}^{2}$, resulting in extremely high values of γ=720 m${}^{-1}$W${}^{-1}$ [7]. Similar to the SOI platform, (Al)GaAs on non-native substrate has been termed (Al)GaAs-OI.

Recently, Lin Chang et al. [196] reported the propagation loss coefficient around 0.4 dB/cm in (Al)GaAs-OI, which is on par with SOI waveguides. Thanks to its superior mode compactness resulting in very high values of γ, and its ultralow propagation loss, (Al)GaAs-OI has been extensively used for fabricating high-Q-factor microring resonators for efficient nonlinear interactions [7,8,119].

### 3.6. Suspended Waveguides

Another group of AlGaAs waveguides with high-refractive-index contrast is suspended waveguides (see Figure 1d,e). Suspended AlGaAs waveguides appear in two forms: a nanowire suspended in the air through lateral tethers and a nanorib waveguide suspended through etch windows [121]. The high refractive index contrast in these waveguides ensures tight light confinement. These waveguide geometries are easy to adapt for modal PM of second-order nonlinear optical interactions.

The specific values of $A_{\mathrm{eff}}$ and γ for suspended waveguides are not reported in the literature. However, based on the known dimensions of these structures [121], the values of γ are expected to be at least as high as those of AlGaAs-OI waveguides.

### 3.7. Multi-Quantum-Well Waveguides

The final group of waveguides, presented in Figure 1g–i, belong to the multilayer heterostructure platform. The majority of waveguide architectures manufactured based on multilayer heterostructures support phase-matched $\chi^{\left(2\right)}$ interactions. Multilayer waveguide structure schematically shown in Figure 1g represents asymmetric quantum well [41,43,44] and selectively-oxidized waveguides [42,46,47,100,107,116,124,128] relying on form birefringence as a PM mechanism.

### 3.8. Orientation-Patterned Waveguides

The orientation-patterned structure shown in Figure 1h schematically represents waveguides with periodical domain disordering (PDD) by quantum-well intermixing (QWI) [110,113,126] and periodical domain inversion (PDI) [30,38,45,104,106,112,127]. These techniques are typically implemented in order to achieve a periodic modulation of $\chi^{\left(2\right)}$, either by domain inversion resulting in the sign change of $\chi^{\left(2\right)}$ or domain disordering resulting in the periodic suppression of $\chi^{\left(2\right)}$. These approaches lead to quasi-phase-matched (QPM) second-order nonlinear optical interactions.

### 3.9. Bragg Reflection Waveguides

Finally, the schematic in Figure 1i shows a waveguide with Bragg reflectors in place of the claddings [108,111,114,123,125]. These reflectors serve as a mechanism for modal phase matching (MPM) in $\chi^{\left(2\right)}$ experiments.

We further outline the common nanofabrication steps used for the fabrication of the majority of the waveguide structures, touching on some specifics concerning more sophisticated waveguide geometries.

## 4. Nanofabrication Approaches

### 4.1. Common Fabrication Steps

The fabrication process of all AlGaAs-based integrated optical devices starts with the epitaxial growth of the desired AlGaAs heterostructure [8,134,197]. The most common sources of optical losses in a waveguide are defects acquired in epitaxial growth. Most typically, these defects are a result of lattice mismatch that can be significant in some semiconductors such as, e.g., GaN [90]. However, as remarked earlier in the manuscript, AlGaAs is lattice-matched for the entire composition range, and there are typically very few defects associated with the process of epitaxy. Molecular beam epitaxy (MBE) or metalorganic chemical vapor deposition (MOCVD) techniques are commonly used for the epitaxial growth of AlGaAs heterostructure wafers.

**Figure 2:**
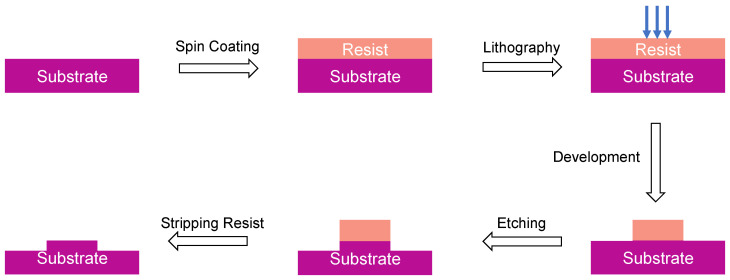
Steps of the top-down (Al)GaAs waveguide fabrication process.

Once the desired AlGaAs heterostructure is ready, one can proceed to fabricating the actual integrated photonic devices, following a top-down fabrication approach. A schematic of this process is shown in Figure 2.

The grown AlGaAs wafer is coated with a photo- or electron-beam (e-beam) resist. Next, the device structures are lithographically patterned into the resist serving as an etching mask for the pattern transfer into AlGaAs. As the next step, selective etching is performed, using an either dry- or wet-etching technique, and the remaining resist is stripped to reveal the final devices.

Dry reactive-ion etching (RIE) process for AlGaAs is typically based on chlorine gas chemistry. It has a selectivity of roughly 1:1 for the photomasks such as ZEP520a. However, dry etching can exhibit the selectivity of up to 1:10 for hard masks such as silica. There have been several studies where the etching of AlGaAs with BCl${}_{3}$ and Cl${}_{2}$ was performed [198,199]. In addition to RIE chemical etching, most reactors have inductively-coupled plasma (ICP) physical etching process accomplished by inertial gases such as Argon. ICP-assisted RIE has an increased etching rate compared to RIE-only process. Some additional studies have shown that adding nitrogen with Cl${}_{2}$ creates a thin passivation layer surrounding an AlGaAs waveguide. Moreover, this approach has been shown to yield a 10-fold increase in the etching rate [200,201].

### 4.2. Suspended Waveguides

To create suspended structures, an additional wet chemical etching step is performed for the removal of the high-aluminum-concentration AlGaAs sacrificial layer [121]. For aluminum concentrations >40%, hydrofluoric acid (HF) is the most common wet etchant. It selectively removes AlGaAs with higher Al concentration while appearing almost non-reactive to lower-Al-concentration layers [202].

### 4.3. PDI Structures

**Figure 3:**
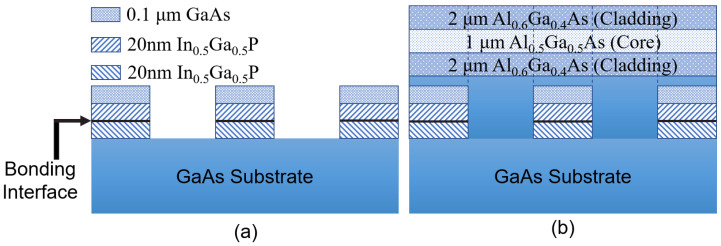
(**a**) Schematic showing a PDI template with varying crystal orientation at the bonding interface and (**b**) the ultimate heterostructure regrown on the patterned PDI template. Adapted from [30], with the permission of AIP Publishing.

A simple, single-step epitaxial process applies to the majority of the waveguide geometries shown in Figure 1, except for PDI waveguides [30,38] schematically represented in Figure 1h. The epitaxial process, in this case, requires a regrowth. First, two separate GaAs substrates are used in order to grow two AlGaAs heterostructures topped with thin layers of InGaP. The latter are bonded with each other so that their [110] crystal directions are parallel to each other, but the domains are reversed at the bonding interface. After the bonding, one of the GaAs substrates and an AlGaAs sacrificial layer are removed by a wet chemical etching process. Grating patterns are then written on the top layer and chemically etched until the lower GaAs substrate is reached (see Figure 3a). The resulting template exhibits a periodic domain inversion. AlGaAs layers are further grown by MOCVD on top of the template such that they form a heterostructure with a higher-index (lower-Al-concentration) core sandwiched between lower-index AlGaAs claddings [see Figure 3b]. As a final step, a waveguide is defined and etched, maintaining PDI in the propagation direction of light.

### 4.4. AlGaAs-OI

**Figure 4:**
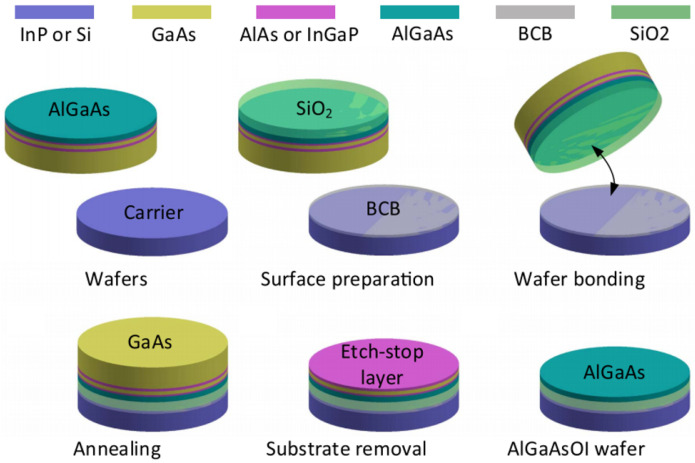
Fabrication process of AlGaAs-OI waveguide platform. Reprinted with permission from [177] ©The Optical Society.

The preparation of an AlGaAs-OI wafer involves bonding an AlGaAs wafer to a carrier InP or Si wafer (see Figure 4 for the entire process). First, AlGaAs epitaxial layers are grown on a GaAs substrate; the grown epitaxy contains some AlGaAs sacrificial etch-stop layers with the actual (Al)GaAs guiding layer at the very top. A layer of SiO${}_{2}$ a few micrometers high is then deposited on top of the (Al)GaAs guiding layer. This SiO${}_{2}$ layer will be the lower cladding in the ultimate (Al)GaAs-OI platform. The as-grown AlGaAs heterostructure coated with SiO${}_{2}$ is then flipped and bonded with the SiO${}_{2}$ side onto a carrier wafer (see Figure 4). This process is followed by sacrificial layers removal through wet chemical etching [8,177]. The prepared AlGaAs-OI wafer is further used for waveguide fabrication, as described in Section 4.1.

## 5. $\chi^{2}$ Nonlinear Phenomena in AlGaAs Waveguides

**Figure 5:**
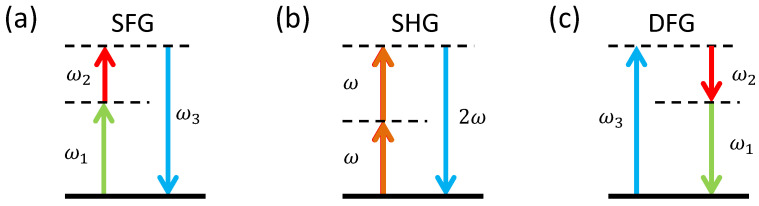
Energy-level diagrams associated with $\chi^{\left(2\right)}$-related phenomena: (**a**) sum-frequency generation, (**b**) second-harmonic generation, and (**c**) difference-frequency generation.

Let us assume that two linearly polarized plane waves with different angular frequencies $\omega_{1}$ and $\omega_{2}$ illuminate a $\chi^{\left(2\right)}$-type (non-centrosymmetric) medium. The total incident electric field can be expressed as $E\left(t\right)=E_{1}e^{-i\omega_{1}t}+E_{2}e^{-i\omega_{2}t}+c.c.$, where $E_{1,2}$ are the electric field amplitudes of the waves. The second-order contribution to the nonlinear polarization, induced by the waves in the medium, can be written as [203]

(3) $$\begin{array}{ccc} P^{\left(2\right)}\left(t\right) & = & \varepsilon_{0}\chi^{\left(2\right)}E^{2}\left(t\right)\\ & = & \varepsilon_{0}\chi^{\left(2\right)}\left(E_{1}^{2}e^{-i2\omega_{1}t}+E_{2}^{2}e^{-i2\omega_{2}t}+2E_{1}E_{2}e^{-i\left(\omega_{1}+\omega_{2}\right)t}+2E_{1}E_{2}^{*}e^{-i\left(\omega_{1}-\omega_{2}\right)t}+c.c.\right)\\ & & +2\varepsilon_{0}\chi^{\left(2\right)}\left(E_{1}E_{1}^{*}+E_{2}E_{2}^{*}\right), \end{array}$$

where $\varepsilon_{0}$ is the vacuum permittivity. As one can see from Equation (3), the polarization generated by the medium in response to the incident optical field contains the contributions oscillating at the sum frequency of the two incident fields (SFG), their difference frequency (DFG), as well as doubled-frequency components, or SHG, of both the fields. The last contribution represents optical rectification; we do not discuss this phenomenon in the manuscript. The energy diagrams representing the three $\chi^{\left(2\right)}$ interactions are shown in Figure 5, where the dashed lines indicate virtual states.

It is evident from Equation (3) that the efficiency of a second-order nonlinear optical process depends on the value of the material’s $\chi^{\left(2\right)}$. Table 3 presents the measured values of the second-order nonlinear susceptibility tensor elements *d* of (Al)GaAs (or their effective values), related to $\chi^{\left(2\right)}$ by $d_{ijk}=\chi_{ijk}^{\left(2\right)}/2$ [203]. SHG is the most used technique for measuring second-order nonlinear coefficients. There are two references in the table that provide crucial information about the dependencies of the nonlinear optical coefficients on other parameters. Ref. [102] reports the measurement of *d* as a function of *x* in ${\mathrm{Al}}_{x}{\mathrm{Ga}}_{1-x}\mathrm{As}$ thin-films and indicates its decrease with an increase in the aluminum fraction *x*. The other work [204] reports the spectral dependence of the second-order nonlinear coefficient and compares it to the relevant theoretical models. Such trends and dependencies are important to consider in the design of integrated optical devices.

The efficiencies of SHG, SFG, and DFG are also affected by the phase mismatch between the interacting waves [203]. For example, the PM requirement for efficient SHG can be written as $\Delta \beta =\beta_{2\omega }-2\beta_{\omega }$, where $\beta_{2\omega }$ and $\beta_{\omega }$ are the propagation constants at the fundamental and second-harmonic frequencies, respectively. Due to the significant difference in the frequencies of the interacting waves, the phase mismatch can be substantial in all these processes, severely compromising their efficiencies. Therefore, in order to realize efficient $\chi^{\left(2\right)}$ nonlinear interactions, it is essential to implement a practical PM scheme.

In a bulk nonlinear medium, PM is performed through natural birefringence attainable only in a handful of nonlinear crystals [206,207,208,209]. In integrated nonlinear photonic devices, PM is accomplished by matching the modal effective refractive indices of the interacting frequency components (MPM) [10,11,14,15,105,109,115,118,119,120,121], selective oxidation for form-birefringence PM [42,46,47,100,107,116,124,128], PM based on Bragg reflectors and gratings [16,108,111,114,123,125], and QPM based on PDI [30,38,45,48,106,112,127], PDD [43,44,110,113,126], and induced directionally [117,122]. We further discuss the most common waveguide PM techniques and present the best results achieved with each of them to date.

### 5.1. Modal Phase Matching

MPM technique has been applied in various AlGaAs platforms and waveguide geometries. Conventionally, it involved equalizing effective refractive indices of lower-frequency fundamental modes and a higher-frequency higher-order mode by a proper waveguide design. Let us consider some specific examples of such a phase-matching arrangement.

**Figure 6:**
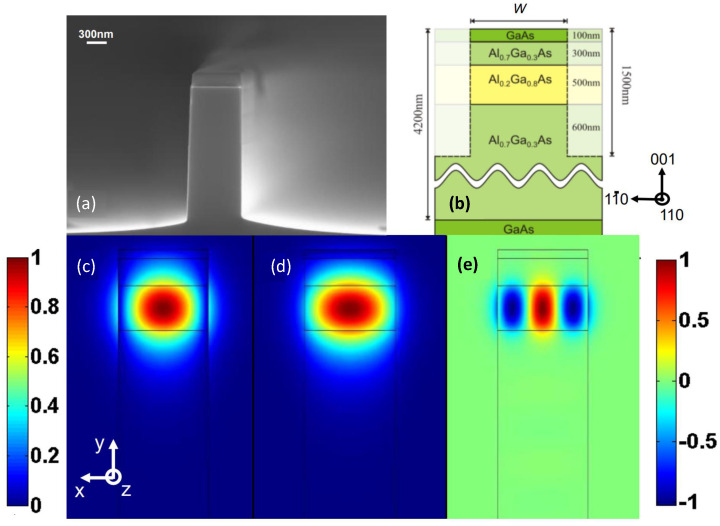
(**a**) Scanning electron microscopy image of an AlGaAs nanowire; (**b**) schematic of the corresponding waveguide geometry. (**c**,**d**) represent TE${}_{00}$ and TM${}_{00}$ modes at the fundamental wavelength $\lambda_{0}=1582$ nm, respectively; (**e**) represents the second-harmonic TE${}_{02}$ mode at $\lambda_{0}=791$ nm. Adapted with permission from [115] ©The Optical Society.

MPM SHG has been realized in AlGaAs nanowires [115], where the fundamental-frequency (FF) TE${}_{00}$ and TM${}_{00}$ modes at $\lambda_{0}=1582$ nm were phase-matched with the second-harmonic (SH) TE${}_{02}$ mode at $\lambda_{0}=791$ nm via a type II PM condition $\Delta \beta =\beta_{\mathrm{FF}1}+\beta_{\mathrm{FF}1}-\beta_{\mathrm{SH}}=\left(n_{\mathrm{FF}1}+n_{\mathrm{FF}2}-2n_{\mathrm{SH}}\right)\omega_{\mathrm{FF}}/c_{0}$, where $n_{i}$ refers to the effective refractive index of mode *i*, and $c_{0}$ is the speed of light in free space. Figure 6 displays a scanning electron microscopy (SEM) image (part (a)) and the schematic (part (b)) of the nanowires from [115]. Parts (c) and (d) of the figure show the simulated fundamental TE${}_{00}$ and TM${}_{00}$ FF modes, and part (e) represents the SH TE${}_{02}$ mode of the structure. The drawback of this PM technique is associated with the modal shape mismatch between different orders of the interacting modes (see Figure 6c–e), resulting in the loss of efficiency.

**Figure 7:**
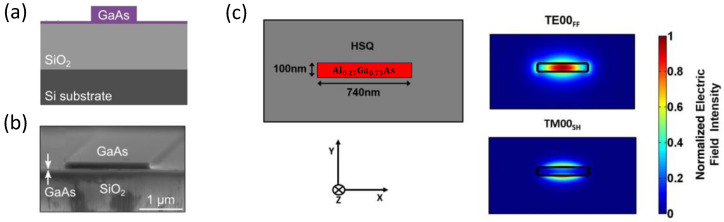
(**a**) Schematic of a GaAs-OI waveguide cross-section. (**b**) SEM image of the waveguide. Reprinted with permission from [10] ©The Optical Society. (**c**) The cross-sectional view of an AlGaAs-OI waveguide and the mode profiles of the fundamental and SH frequencies (with orthogonal polarizations) fulfilling MPM. Reprinted with permission from [11] ©The Optical Society.

Another example is illustrated in Figure 7 where GaAs-OI [10] (see Figure 7a,b) and AlGaAs-OI [11] (see Figure 7c) waveguides are displayed. The high asymmetry of the guiding channel creates modal birefringence between the two orthogonal fundamental modes. This birefringence is sufficient to achieve phase-matching between the FF TE${}_{00}$ and the SH TM${}_{00}$ modes. SHG with MPM through modal birefringence in highly asymmetric waveguide cross-section has also been realized in suspended nanowire and nanorib waveguides [121]. The work by E. J. Stanton et al. featured in Figure 7a–c reports the highest SHG conversion efficiency of 47,000%/(W cm${}^{2}$) obtained in an (Al)GaAs waveguide to date [10].

The benefit of MPM technique resides in its simplicity of the implementation. MPM devices do not require any sophisticated fabrication procedures; as a minimum, it is sufficient to implement a single-step epitaxy and a single-step lithography and etching to fabricate such a device [115].

### 5.2. Phase Matching in Bragg Reflection Waveguides

**Figure 8:**
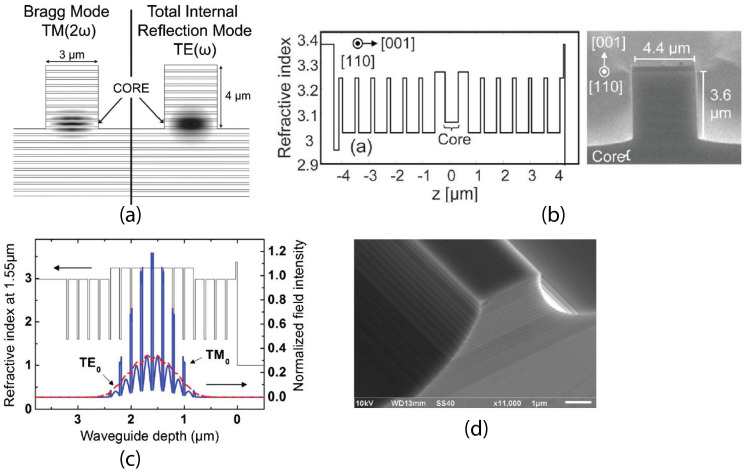
(**a**) Intensity profiles of the phase-matched fundamental TM${}_{00}$ (SH) and TE${}_{00}$ (FF) modes of a BRW. Reproduced from [108], with the permission of AIP Publishing. (**b**) Refractive index profile of a BRW in vertical dimension and an SEM image of a BRW cross-section. Adapted with permission from [123] ©The Optical Society. (**c**) Refractive index (thin solid line) and intensity profiles of the TE-polarized FF (thick solid line) and TM-polarized SH (dashed line) modes in a selectively oxidised waveguide. Reprinted with permission from [100] ©The Optical Society. (**d**) SEM image of a selectively oxidized waveguide with form birefringence. Reprinted with permission from [100] ©The Optical Society.

Bragg reflection waveguides (BRW) represent a special type of MPM structure that deserves separate attention. They utilize a structure that can support guided modes via both the total internal reflection (TIR) and transverse Bragg reflection [108,111,114,123,125,193]. The latter is achieved by the periodic structures (Bragg reflectors) defined above and below the guiding layer, see Figure 1i and Figure 8b. Specifically, strong modal dispersion of a photonic bandgap device is exploited to achieve a PM between the fundamental modes guided by different mechanisms and carrying light at different optical frequencies. Typically, a lower-frequency fundamental TIR mode is phase-matched with the higher-frequency fundamental Bragg mode (see Figure 8a,b). In such a way, BRW offer PM between the lowest-order modes of the involved frequencies. This allows for a maximum modal field overlap and optimal pump power utilization.

An additional benefit of BRW nonlinear optical waveguides is the fact that they are readily integratable with semiconductor laser technologies for monolithic integration of a diode laser with a nonlinear frequency converter [111]. This feature makes such a scheme easily realizable in practice.

### 5.3. Form Birefringence Phase Matching

Exact PM can also be achieved in a waveguide with form birefringence [42,46,47,100,107,116,124,128]. Such waveguides are made of alternating subwavelength layers of high-index AlGaAs and low-index aluminum oxide AlO${}_{x}$ (see Figure 8c,d). A starting material is a multilayer stack of (Al)GaAs/AlAs alternating layers. As is, such an arrangement does not have sufficient birefringence for phase matching $\chi^{\left(2\right)}$-type nonlinear interactions, and it has been proposed [42,46] to apply post-growth selective wet oxidation to convert the original stack into (Al)GaAs/AlO${}_{x}$. Thanks to the large refractive index difference between (Al)GaAs and AlO${}_{x}$ ($\Delta n_{\max}=2$), it becomes possible to achieve a sufficient birefringence for PM second-order nonlinear interactions. The lower- and higher-frequency beams with the polarizations parallel and perpendicular to the layer interfaces can experience similar effective refractive indices [100] (see Figure 8c).

It is worthwhile noticing that some MPM suspended [121] and (Al)GaAs-OI [10,11] waveguides also exhibit birefringence due to the asymmetry in the shape of their guiding channels. As such, the highest efficiency in birefringence-PM AlGaAs waveguides was reached in an AlGaAs-OI waveguide [10], thanks to its superior compactness and ultralow propagation loss.

### 5.4. Quasi Phase Matching

Besides MPM and BPM, there were numerous QPM schemes proposed. QPM techniques include periodic reversal of the asymmetry of quantum wells and other periodic half-wavelength structures for surface-emitted SHG [37,41], QPM via a periodic corrugation of the surface of a waveguide [16] and numerous orientation-patterning techniques. Among the latter, there are periodic domain inversion [30,38,45,48,106,112,127,210], and periodic $\chi^{\left(2\right)}$ suppression and reduction in asymmetric coupled quantum well (ACQW) waveguides [43,44] and domain disordering via quantum-well intermixing (QWI) [110,113,126,211,212]. Another kind of QPM, an orientational QPM, has been recently demonstrated in snake-shaped suspended nanowires, producing QPM SHG [117]. The majority of QPM SHG waveguides are based on PDI and PDD structures. We find it noteworthy to describe such structures in more detail, highlighting their similarities and distinctions.

**Figure 9:**
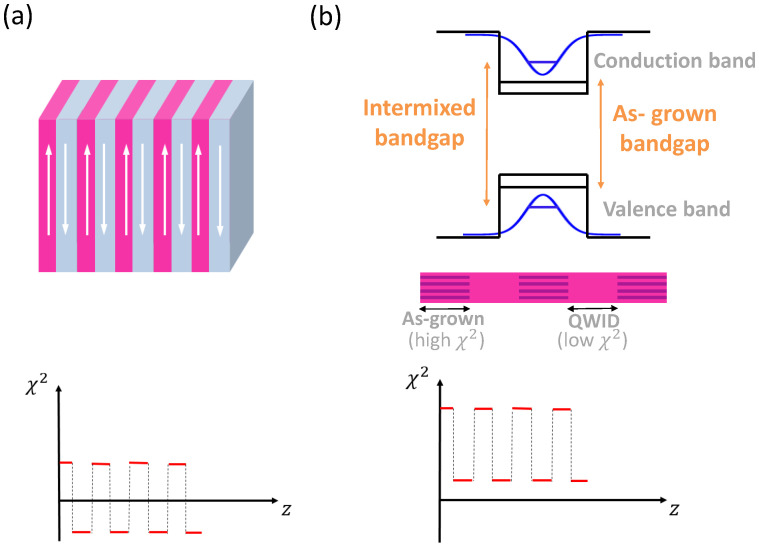
(**a**) The top part shows a schematic of a PDI QPM structure. The crystalline orientation is periodically altered with respect to <001> axis. The sign of $\chi^{\left(2\right)}$ is, consequently, flipped, as schematically shown in the bottom part. (**b**) Top: energy-band diagram of GaAs/AlGaAs quantum well, as-grown and intermixed. The process of QWI results in an increase in the energy gap compared to that of an as-grown structure, which results in a decrease in the value of $\chi^{\left(2\right)}$. The drawing in the middle shows the longitudinal cross-section of a PDD waveguide having as-grown and QWI domains (QWID). The bottom portion schematically shows the impact of the PDD through QWI on the values of $\chi^{\left(2\right)}$: it becomes periodically suppressed in the QWID regions.

In the PDI structures, QPM is obtained through a periodic domain inversion, resulting in the sign change of $\chi^{\left(2\right)}$ (see Figure 9a). Using this technique, S. Wang et al. demonstrated two orders of magnitude DFG conversion-efficiency enhancement compared to that achievable in LiNbO${}_{3}$ waveguides [127]. Waveguides with domain inversion can be fabricated by using an orientation-patterned substrate as a template (OP-QPM) [210], see also Section 4.3.

In PDD waveguides, the $\chi^{\left(2\right)}$ of the core made of multiple GaAs/AlGaAs quantum wells is periodically modulated in the propagation direction through domain disordering via QWI by ion implantation [110,113,126]. As a result, as-grown (non-intermixed) structures have a higher $\chi^{\left(2\right)}$, while intermixed structures have their $\chi^{\left(2\right)}$ suppressed due to domain disordering. In Figure 9b (top portion), we schematically show how QWI affects the band-gap energy of AlGaAs heterostructure. Specifically, QWI increases the energy gap, which leads to a decrease in the associated value of $\chi^{\left(2\right)}$. Furthermore, QWI is performed in a periodic manner through a lithographically defined gold mask [110], so that the resulting structure has domains with the values of $\chi^{\left(2\right)}$ alternating between high (as-grown, or non-intermixed QW structure) and low (QWI structure), see the middle portion of Figure 9b. As a result, PDD structures exhibit domains with different values of $\chi^{\left(2\right)}$, alternating between high and low values without a sign reversal (see the bottom portion of Figure 9b). In contrast, in PDI structures, we are dealing with a periodic sign reversal of $\chi^{\left(2\right)}$ due to the crystalline domain inversion (see the bottom part of Figure 9a). Other than this distinction, both PDI and PDD QPM structures have a similar principle of operation, based on a periodic modulation of $\chi^{\left(2\right)}$ values.

We covered a variety of PM techniques implemented in (Al)GaAs waveguides to enhance second-order nonlinear interactions. All these PM schemes and technologies have been improving over the years, and a natural question arises as to which PM scheme results in the most efficient $\chi^{\left(2\right)}$ interactions. In the following section, we answer this question by summarizing the phase-matching techniques and the corresponding $\chi^{\left(2\right)}$ processes’ conversion efficiencies in a table.

### 5.5. Summary of $\chi^{\left(2\right)}$ Waveguide Performances

In Table 4, we present the best performing (Al)GaAs devices with different phase-matching approaches for SFG, DFG, and SHG second-order nonlinear effects. The table is organized by grouping the works based on the nonlinear effect, presenting SFG followed by DFG and SHG below. For each nonlinear effect, the works with the highest conversion efficiencies are presented closer to the top. We specify the material platform, waveguide geometry, PM mechanism, incident and generated wavelengths, and conversion efficiency for each work.

For a long time, QPM by PDI and PDD has been a popular approach for achieving enhanced $\chi^{\left(2\right)}$ interactions in AlGaAs waveguides. The reason for interest in these techniques was a relatively high conversion efficiency reported in the early works. The competing techniques have been form-birefringence waveguides with selective oxidation and BRW. All these PM techniques have been developing over the years; they all have seen significant improvements in the associated propagation losses and conversion efficiencies.

One of the shortcomings of many of these approaches is the fabrication complexity. Some of the structures above require multistep fabrication processes. For example, PDI waveguides require epitaxial regrowth or complex template patterning procedures, PDD structures require two-step lithography and ion implantation, and waveguides with oxide layers and form birefringence require wet oxidation. In the end, the best-performing PDI, PDD, and selective-oxidation devices feature comparable normalized SHG conversion efficiencies on the order of 1000 %/(W cm${}^{2}$).

BRW waveguides represent a step forward in many aspects, including the simplicity of fabrication (they require a single-step epitaxy and lithography), integrability with on-chip light sources, and an order-of-magnitude improvement in conversion efficiencies of SHG processes compared to those demonstrated in the early PDI, PDD, and selective-oxidation works. However, a true breakthrough was possible thanks to the recent demonstrations of $\chi^{\left(2\right)}$ interactions in ultra-low-loss suspended [121] and GaAs-OI [10] waveguides exhibiting very high conversion efficiencies. These works designate a new era in AlGaAs nonlinear integrated photonics—where device simplicity and operational efficiency come together.

## 6. $\chi^{\left(3\right)}$ and $\chi^{\left(5\right)}$ Nonlinear Phenomena in AlGaAs Waveguides

The third- (and especially fifth-) order contributions to the nonlinear optical polarization coming from the third-order nonlinear susceptibility $\chi^{\left(3\right)}$ and fifth-order susceptibility $\chi^{\left(5\right)}$, respectively contain very many different terms describing a variety of nonlinear optical effects [203]. In this manuscript, we concentrate only on the third- and fifth-order nonlinear effects most studied in AlGaAs optical waveguides. Among these third-order effects, there are FWM [7,9,20,132,133,134,135,136,137,138,139,140,141,142,143,144], 2PA [13,19,23,114,145,146,147,148], SPM [23,32,114,133,134,146,147,149,150,213], XPM [32,133,146,149], SFWM [151,152], SCG [154,155], and Kerr frequency microcomb [8,156]. The $\chi^{\left(5\right)}$-induced 3PA [13,19,27,134], which represents an important factor in AlGaAs waveguide nonlinear optical experiments, will also be briefly discussed in this section.

### 6.1. Nonlinear Refraction

The overall expression for the refractive index of a nonlinear optical medium containing both the linear and nonlinear contributions has the form

(4) $$n=n_{0}+n_{2}I,$$

where $n_{0}$ is the linear refractive index, $n_{2}$ is the Kerr coefficient associated with $\chi^{\left(3\right)}$, as discussed above, and *I* is the intensity of light. The intensity-dependent contribution $n_{2}I$ entering the Equation (4) for *n* is responsible for many nonlinear optical effects such as SPM, XPM, FWM, and some others. In Table 5, we consolidate the experimental values of $n_{2}$ for (Al)GaAs waveguides of different platforms and geometries, with different guiding layer compositions. The value for bulk GaAs is also presented in the table for comparison. The guiding layers of multi-quantum-well waveguides are made of multiple GaAs wells with ${\mathrm{Al}}_{x}{\mathrm{Ga}}_{1-x}\mathrm{As}$ barriers, with *x* ranging from 0.2 to 0.4 (or up to 1 for superlattice waveguides). For the 2- or 3-layer platforms, the aluminum content at the core region ranges from x=0.14 to 0.26, but the composition x=0.18 is of particular interest because it corresponds to the minimum of nonlinear absorption losses at 1550nm resulting from a favorable trade-off between the values of 2PA and 3PA coefficients, as we discuss further in the next subsections. The Kerr coefficient for x=0.18 remains high at the expense of the minimal nonlinear loss. Additionally, $n_{2}$ decreases with an increase in the aluminum content *x* (and the corresponding bandgap energy) of the guiding layer [135].

One of the nonlinear optical parameters associated with $n_{2}$ is the nonlinear phase shift $\phi^{\mathrm{NL}}$ accumulated by an optical wave as it propagates through a waveguide of length *L*, which can be expressed as [28]

(5) $$\phi^{\mathrm{NL}}=\frac{2\pi }{\lambda_{0}}n_{2}\int_{0}^{L}I\left(z\right)dz.$$

This parameter is crucial for evaluating the potential of an optical device for nonlinear switching applications. It has been determined that a waveguide needs to demonstrate $\phi^{\mathrm{NL}}$ over 2π in order to meet the requirements for all-optical switching [28,214]. It has also been shown that a nonlinear phase shift in excess of 5π is readily achievable in AlGaAs [133,134,213]. However, the large nonlinear phase shift is not the only requirement for a material to be deemed as suitable for nonlinear switching application. An important role plays the overall absorption and speed of the nonlinearity. We further comment on these characteristics of AlGaAs.

### 6.2. Nonlinear Absorption and Figures of Merit

The overall absorption in a nonlinear material, including linear and nonlinear contributions, can be written as [28,147]

(6) $$\alpha =\alpha_{0}+\alpha_{2}I+\alpha_{3}I^{2},$$

where $\alpha_{0}$, $\alpha_{2}$, and $\alpha_{3}$ are the linear absorption, 2PA, and 3PA coefficients, respectively. The overall absorption is highly wavelength-dependent; therefore, further analysis depends on the photon energies considered. Additionally, the optical field of the guided mode penetrates to the cladding regions in different waveguide geometries to a different extent. Hence, the measured nonlinear coefficient represents an intensity-weighted average over the different layers of the waveguide structure [148,215]. In Table 6, we summarize the values of $\alpha_{2}$ and $\alpha_{3}$ measured in waveguides of various compositions, platforms, and geometries, together with some bulk-material values for comparison.

AlGaAs at the photon energies below the bandgap has small linear absorption and fast bound-electron nonlinearity with sub-picosecond response [28]. However, additional considerations reside in the values of nonlinear losses coming from multi-photon absorption. The specific parameters, known as nonlinear figures of merit (FOM), were introduced in order to identify a suitable wavelength range for optical switching in AlGaAs integrated devices [28]. We further introduce these parameters.

Let us first consider the case where the linear absorption is very small, but the 2PA is the dominant loss mechanism. This situation arises at the photon energies smaller than the bandgap but higher than or comparable to half-the-bandgap. Thus, the intensity of light propagating through the waveguide varies according to the following equation:

(7) $$\frac{dI}{dz}=\alpha_{0}I+\alpha_{2}I^{2}.$$

The solution to the above equation can be written as

(8) $$I\left(z\right)=I\left(0\right)\frac{1}{\exp\left(\alpha_{0}z\right)\left\{1+\frac{\alpha_{2}}{\alpha_{0}}I\left(0\right)\left[1-\exp\left(-\alpha_{0}z\right)\right]\right\}}.$$

Substituting Equation (8) into Equation (5), we can find the value for the nonlinear phase shift under 2PA conditions:

(9) $$\phi^{\mathrm{NL}}=\frac{2\pi }{\lambda_{0}\alpha_{2}}n_{2}\ln\left[1+\alpha_{2}I\left(0\right)L\right].$$

Further, the requirement $\phi^{\mathrm{NL}}>2\pi$ translates into

(10) $${\mathrm{FOM}}_{2\mathrm{PA}}=\frac{\lambda_{0}\alpha_{2}}{n_{2}}<1,$$

where ${\mathrm{FOM}}_{2\mathrm{PA}}$ is the FOM associated with 2PA as the limiting effect.

Under the conditions where 2PA is weak (for the photon energies far below half-the-band-gap), a situation may arise where 3PA is the dominant loss mechanism [27]. It is, therefore, instructive to introduce another FOM related to 3PA [13]:

(11) $${\mathrm{FOM}}_{3\mathrm{PA}}=\frac{\lambda^{2}\alpha_{3}}{n_{2}L_{c}}\frac{A_{\mathrm{eff}}^{\left(3\right)}}{{\left(A_{\mathrm{eff}}^{\left(5\right)}\right)}^{2}},$$

where $L_{c}$ is the characteristic length (equal to one half beat length), $A_{\mathrm{eff}}^{\left(3\right)}$ and $A_{\mathrm{eff}}^{\left(5\right)}$ represent the effective mode areas for the third- and fifth-order nonlinear process, respectively. It has been demonstrated that for a practical situation, the requirement ${\mathrm{FOM}}_{3\mathrm{PA}}<1$ needs to be fulfilled [218]. In such a way, the nonlinear FOMs given by Equations (10) and (11) yield the parameter space for practical nonlinear switching devices. It has been shown that AlGaAs readily satisfies these requirements for the photon energies below, but is still close to half-the-bandgap [28].

Generally, a practical consideration for compact on-chip all-optical devices operating at the Telecom C-band is to select the material composition of Al${}_{x}$Ga${}_{1-x}$As in a way that the bandgap is above 750 nm. This requirement is satisfied by selecting Al${}_{0.18}$Ga${}_{0.82}$As as the composition of the guiding layer in integrated optical devices [28]. The composition can be modified to target a different wavelength range as required by an application.

Nonlinear absorption, however, is not a purely detrimental effect. In [145], the authors demonstrate all-optical logic gates that operate based on 2PA in AlGaAs microring resonators.

In practice, it is essential to have the information about wavelength dependence of the nonlinear refraction coefficient $n_{2}$ as well as 2PA and 3PA coefficients $\alpha_{2}$ and $\alpha_{3}$ for the specific material compositions considered in nonlinear optical devices. Some of these measurements have been performed; we summarize these studies below.

### 6.3. Dispersion of $n_{2}$ and Nonlinear Absorption Coefficients

The wavelength dependencies of $n_{2}$ and 2PA coefficient $\alpha_{2}$ near half-the-bandgap were first measured in [23]. The study of the wavelength dependence of the 3PA coefficient $\alpha_{3}$ has been performed for the photon energies between half-the-bandgap ($E_{g}/2$) and 1/3 of the bandgap energy ($E_{g}/3$) for Al${}_{x}$Ga${}_{1-x}$As with x=0.18, 0.19, and 0.20 [27]. The dispersion of $n_{2}$, $\alpha_{2}$, and $\alpha_{3}$ was also measured in [13], where the authors gave a special consideration to the polarization-dependent measurements and the comparison with the existing theoretical models [219,220]. Nonlinear spectroscopy and polarization dependencies of $n_{2}$ and $\alpha_{2}$ near $E_{g}/2$ have also been performed in bulk and quantum-well AlGaAs ridge waveguides in [19].

**Figure 10:**
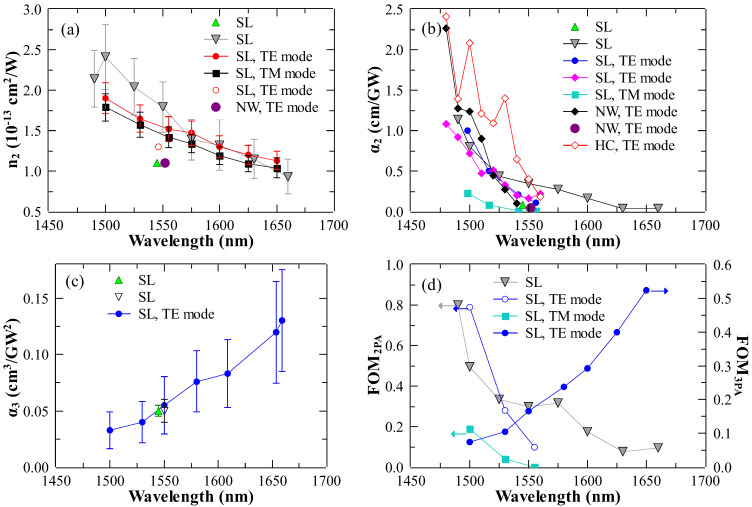
The spectra of the nonlinear coefficients of AlGaAs waveguides with ${\mathrm{Al}}_{0.18}{\mathrm{Ga}}_{0.82}\mathrm{As}$ guiding layer: (**a**) nonlinear refractive index, (**b**) 2PA coefficient, (**c**) 3PA coefficient, and (**d**) nonlinear FOM associated with 2PA and 3PA, as defined by Equations (10) and (11). References: • [9]; •, *▪*, and ∘ [13]; ▴ [22]; ▾ [23]; ▿ [28]; ∘ [36]; • and *▪* [39]; ⧫, *◊*, and ⧫ [148].

The spectra of the nonlinear optical coefficients of waveguides with ${\mathrm{Al}}_{0.18}{\mathrm{Ga}}_{0.82}\mathrm{As}$ guiding layer are shown in Figure 10. Parts (a), (b), and (c) of the figure show the wavelength dependencies of $n_{2}$, $\alpha_{2}$, and $\alpha_{3}$, respectively. The trade-off between the nonlinear loss and nonlinear refraction can be better expressed through the nonlinear FOM (see Equations (10) and (11)), presented in Figure 10d. The smaller the FOM is, the lower the nonlinear loss relative to the nonlinear refraction. We can observe from Figure 10d (TE mode) that FOM${}_{2\mathrm{PA}}$ decreases with wavelength, while FOM${}_{3\mathrm{PA}}$ increases with wavelength. The region where both FOM${}_{2\mathrm{PA}}$ and FOM${}_{3\mathrm{PA}}$ are acceptably small falls in the range of 1550nm. Therefore, Al${}_{x}$Ga${}_{1-x}$As with x=0.18 was selected as the guiding layer for low-loss integrated nonlinear photonic devices operating at 1550nm.

A significant anisotropy of $n_{2}$ and $\alpha_{2}$ has been experimentally observed in [146] in AlGaAs superlattice waveguide with the core made of 14:14 GaAs/AlAs monolayers. In the follow-up study [150], the authors demonstrate how quantum-well intermixing by ion implantation can be implemented to control the values of $n_{2}$ in AlGaAs superlattice waveguides. Specifically, a significant reduction in $n_{2}$ by up to 71%, accompanied by a bandgap energy blue-shift by 68 nm, has been achieved in QWI samples in comparison to as-grown (non-intermixed) superlattice waveguides.

Nonlinear refraction and absorption spectroscopy in mid-IR has been performed in bulk GaAs wavers in [157]. The goal of the study was to establish the impact of 2PA, 3PA, and FCA at longer-wavelength range targeted by some new applications.

Measuring the dispersion of the nonlinear parameters for the entire range of AlGaAs material compositions is a very extensive task requiring a lot of resources. It is more practical by far to rely on the existing theories capable of predicting these values from the band structure of a zinc-blende semiconductor [219,220,221,222,223,224,225] and some associated parameters such as its bandgap energy $E_{g}$. For more detail on these theories, we refer the reader to Appendix A.

### 6.4. $n_{2}$-Related Nonlinear Optical Phenomena

**Figure 11:**
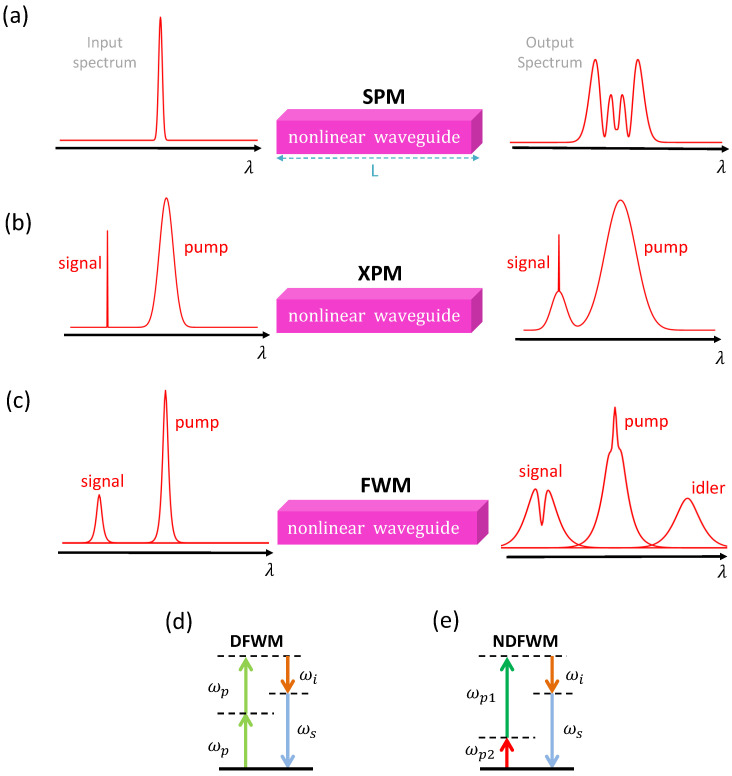
Spectral representations of some $n_{2}$-related nonlinear optical phenomena: (**a**) SPM, (**b**) XPM, and (**c**) FWM. The signal spectrum may appear broadened due to the SPM and XPM effects. The energy diagrams represent (**d**) degenerate FWM (DFWM) and (**e**) non-degenerate FWM (NDFWM).

Let us consider a situation where two beams of light with distinct frequencies are coupled into an optical waveguide having a high value of $n_{2}$. This arrangement typically results in several simultaneous nonlinear optical phenomena that one can observe in the spectrum of the output radiation after it passes through the waveguide. They are illustrated in Figure 11 and described below.

First of all, each optical beam with a sufficient intensity undergoes SPM. The nonlinear phase shift experienced by the high-intensity beam propagating through the waveguide results in the beam’s spectral broadening and, in some circumstances, the appearance of additional peaks in its spectrum (see Figure 11a). SPM is crucial for optical switching, wavelength conversion, and frequency-comb generation. Furthermore, SPM experiments were conducted to measure the effective value of $n_{2}$ in AlGaAs waveguides [146,150]. SPM with a nonlinear phase shift of >5π has been demonstrated in AlGaAs strip-loaded waveguides [133,134,213]. Further, in [147], a slow-light-induced SPM enhancement in air-bridge AlGaAs photonic crystal slab waveguide has been demonstrated.

The second phenomenon that can be observed in the spectra of the output waves is XPM. The intensive waves can act on each other while propagating through the nonlinear waveguide medium, inducing spectral broadening of each other’s spectrum. The mechanism is similar to SPM, except that the waves modify the propagation conditions for each other by changing the overall refractive index of the waveguide medium by the intensity-dependent contribution $n_{2}I$ to the overall refractive index (see Equation (4)). The effect is illustrated in Figure 11b where the incident radiation is comprised of a pulsed pump and a continuous-wave (CW) signal. In this particular case, the more powerful beam (the pump) modifies the signal’s spectrum. XPM has been implemented in wavelength conversion experiments [197,226]. XPM and SPM have been studied in AlGaAs nanowires [149] and 1D slab waveguides [32]. The latter work has demonstrated that a unity ratio of SPM/XPM is achievable in AlGaAs—the condition necessary for spatial-soliton observation.

The third phenomenon that can occur is the generation of an additional spectral component through the FWM process (see Figure 11c). This component can be registered in the output spectrum; it is termed the idler. The frequency $\omega_{i}$ of the idler is related to the pump and signal frequencies ($\omega_{p}$ and $\omega_{s}$, respectively) according to $\omega_{i}=2\omega_{p}-\omega_{s}$. This effect is called degenerate FWM, or DFWM (see the corresponding energy diagram in Figure 11d). In some situations, two distinct powerful pump sources with different frequencies $\omega_{p1}$ and $\omega_{p2}$ and a signal source coupled into the waveguide. In that case, the FWM interaction becomes non-degenerate (NDFWM), and the corresponding energy conservation law is $\omega_{i}=\omega_{p1}+\omega_{p2}-\omega_{s}$ (see the energy diagram in Figure 11e). FWM, both degenerate [7,9,132,133,134,135,136,137,139,140,141,142,143,144,158] and non-degenerate [20], has been extensively studied in AlGaAs waveguides. This effect represents a special importance for all-optical wavelength conversion and signal processing.

The conversion efficiency of the FWM process, defined as the idler-to-signal power ratio (where the signal is typically considered at the input to the nonlinear waveguide), is determined by the pump intensity, nonlinearity of the waveguide, phase mismatch, overall losses experienced by the light in the waveguide, and the interaction length inside the waveguide. Remarkably, phase-matching FWM is generally not as challenging as phase-matching second-order nonlinear phenomena because the frequency difference of the interacting waves in FWM can be very insignificant. Furthermore, for the short propagation lengths associated with optical waveguides, even the presence of a non-zero phase mismatch does not compromise the observation of efficient tunable FWM [134,137,144]. That is why the waveguide geometries for $n_{2}$-related third-order nonlinear interactions are much simpler, and the associated fabrication tolerances are much more relaxed. Nevertheless, many AlGaAs platforms and waveguide geometries permit dispersion engineering for the state-of-the-art performance in enhanced ultra-broad-band FWM, SPM and supercontinuum generation. We further summarize works reporting the highest conversion efficiencies and widest conversion bands of FWM in AlGaAs waveguides (see Section 6.5), and finish this section by highlighting the state-of-the-art AlGaAs devices based on $\chi^{\left(3\right)}$ interactions (Section 6.6).

### 6.5. FWM Conversion Efficiencies

**Figure 12:**
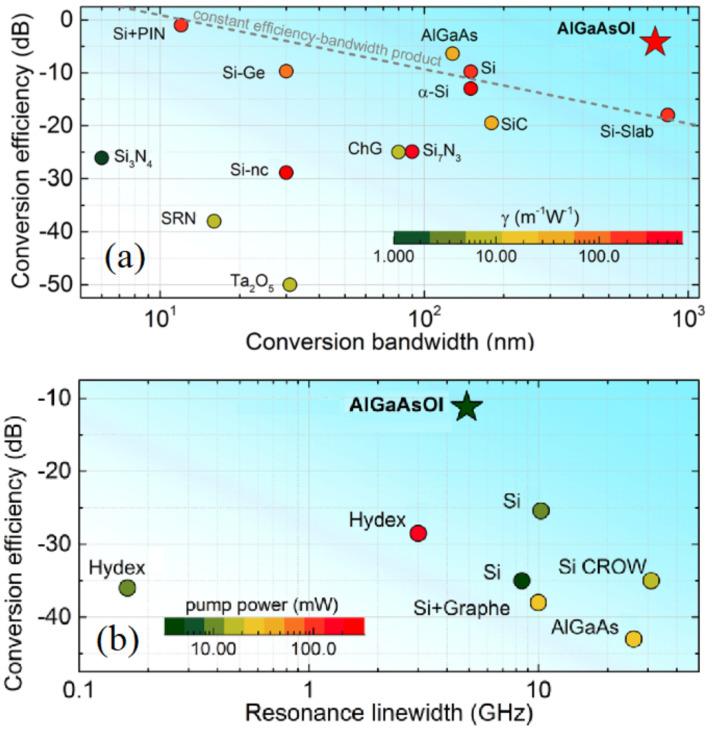
FWM conversion efficiency reported in (**a**) waveguides and (**b**) microring resonators fabricated in different material platforms. This figure was adapted with permission from Ref. [140] whose results are marked with the stars on the graphs. The works reporting other results marked with circles can be found in Ref. [140].

Figure 12a,b displays the best-achieved FWM conversion efficiencies in numerous waveguide and MRR material platforms, respectively. It can be seen from the figures that AlGaAs-OI waveguide platform demonstrates the best FWM performance in terms of both the conversion efficiency and bandwidth [140].

In Table 7, we summarize the highest FWM conversion efficiencies achieved in different AlGaAs waveguide platforms reported to date. We report the results obtained both in waveguides and in MRR, separating them into two corresponding groups. We arrange the results from the highest to the lowest conversion efficiency within each group out of the highest reported values. It is evident from the table that the highest FWM conversion efficiency reported to date has been achieved in AlGaAs-OI.

### 6.6. State-of-the-Art in AlGaAs $\chi^{\left(3\right)}$ Research

In this section, we overview the state-of-the-art in AlGaAs integrated photonic devices operating based on $\chi^{\left(3\right)}$ interactions. As remarked in the preceding section, AlGaAs-OI has been demonstrating the best nonlinear optical performance compared to other AlGaAs waveguide platforms. Furthermore, the superior nonlinear optical performance of AlGaAs-OI also made possible a successful demonstration of supercontinuum generation (SCG) and frequency-comb generation (FCG) in this waveguide platform. Therefore, this section is dedicated to an overview of these latest achievements.

**Figure 13:**
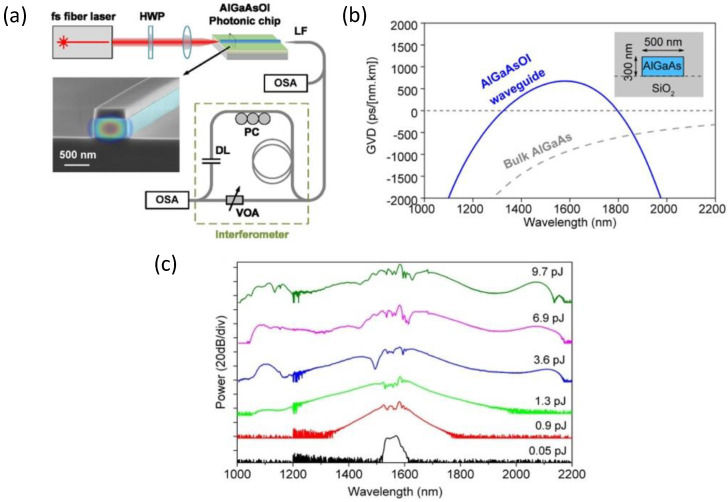
Octave-spanning SCG in AlGaAs-OI platform. (**a**) Schematic of the experimental setup for the spectra measurement. The inset shows an SEM image of the waveguide and the simulated mode profile inside the core. (**b**) Engineered GVD exhibiting anomalous dispersion in the pump wavelength range. (**c**) SCG spectra in AlGaAs-OI waveguide (with the 500-nm width) at different input pulse energies. The 3PA effect causes the saturation of the spectrum broadening with the input pulse power increase. Adapted with permission from [154] ©The Optical Society.

SCG sources are light sources with extremely broad spectra. As a result, they typically cover a broader spectrum than tunable lasers, and they find applications as tunable light sources in spectroscopy. Their other applications include microscopy, optical communication networks, metrology and characterization of optical devices. Supercontinuum is generated by launching a series of ultrashort pulses into a dispersion-engineered waveguide; the underlying nonlinear mechanism is SPM. Efficient SCG requires engineering of higher-order dispersion in waveguides [194,227]. A broadband, octave-spanning SCG with a pulsed laser at the Telecom wavelength in an AlGaAs-OI waveguide has been reported by B. Kuyken et al. [154], see Figure 13.

The main advantage of using AlGaAs-OI waveguide platform for SCG is the possibility for dispersion engineering, resulting in low (to avoid the temporal walk-off) and flat (to suppress the third-order dispersion) GVD, facilitating the formation of a broadband SCG [155]. Another advantage is the possibility for integration of an AlGaAs-based *f*-to-2f interferometer with an SCG source on a single chip. This device is essential for optical metrology, and it requires a $\chi^{\left(2\right)}$ material platform for operation. AlGaAs satisfies this requirement.

**Figure 14:**
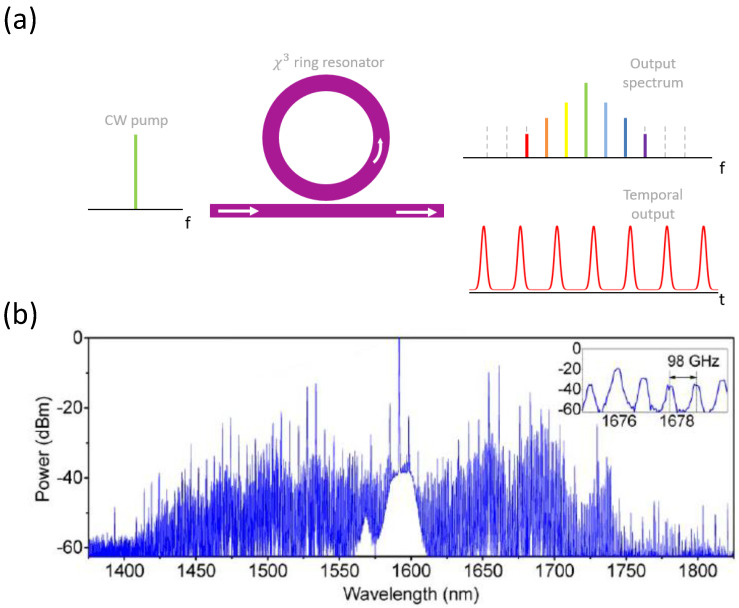
Kerr frequency microcomb generation in an AlGaAs-OI MRR. (**a**) Schematic illustration of the basic idea: an input spectrum, an MRR, and an output spectrum and temporal output. The grey dashed lines in the output spectrum show the MRR frequency modes. The temporal output shows successive pulses (solitons). (**b**) Kerr frequency comb spectrum generated in an AlGaAs-OI MRR driven by 72 mW of CW pump power at 1590 nm. The spectral distance between the lines is 98 GHz. Adapted with permission from [8] ©The Optical Society.

MRR can exhibit a significant enhancement of nonlinear optical interactions at lower incident power levels compared to straight waveguides. Such a device can include a ring and one or two bus waveguides for coupling in and out (see Figure 14a for the schematic). The microring itself shows a resonant behavior based on the constructive interference of the light incoming from the bus waveguide and the light already coupled into the ring. Minimization of the linear loss of the device results in higher values of its quality (Q)-factor and is critical for achieving enhanced nonlinear interactions at minimal power levels. Another essential geometrical feature of an MRR is the coupling distance between the ring and the bus waveguide (see Figure 14a). The different choices of this distance lead to different coupling regimes, known as critical coupling, undercoupling, and overcoupling, as required by a particular application. The former is achieved when the transmission through the through port vanishes and all the incoming light exits through the drop port. The two latter examples are associated with a nonzero transmission with higher and lower Q-factors, respectively, compared to that of the critical coupling, and with different phases. AlGaAs waveguide platforms meet the favorable conditions in terms of linear loss, selection of coupling regime and dispersion engineering.

One of the first demonstrations of FWM in an AlGaAs ring resonator was performed by P. Kultavewuti et al. in a deeply-etched AlGaAs nanowire ring resonator with Q=7500 [138]. Several follow-up studies have been performed, reporting an increase in conversion efficiencies by improving the Q-factor. These studies primarily used AlGaAs-OI MRR [8,196,228].

FWM is an underlying mechanism for Kerr FCG. Kerr frequency microcombs (based on high-Q ring resonators) are on-chip photonic light sources that produce a spectrum with precisely equally spaced spectral lines, finding application in optical communication networks, spectroscopy, optical clocks, radiofrequency photonics, and other fields [229]. They have very compact footprints and low driving power (a few mW or less) due to the inverse quadratic dependence of their threshold power on the Q-factor, $P_{\mathrm{th}}∼A_{\mathrm{eff}}/\left(n_{2}Q_{t}^{2}\right)$, where $Q_{t}$ represents the total quality factor. The initial Kerr frequency peaks can be generated by a DFWM process that uses two pump photons with identical frequencies. At a sufficient power level, a cascaded FWM effect can result in sideband generation through a nondegenerate process, i.e., through the incorporation of two photons with different frequencies to create two new spectral components with up- and down-shifted frequencies. In a high-Q MRR with well-engineered dispersion, the process can continue in a cascaded manner, giving rise to more and more newly generated peaks in the output spectrum.

The temporal dynamics of a frequency microcomb depends on an interplay of various factors [230]. For example, the parametric gain and loss and the nonlinearity and dispersion can play balancing and controlling roles in the pulse dynamics. The temporal dynamics can be described by a nonlinear partial differential equation known as Lugiato–Lefever equation (LLE) [230]. One possible solution to the LLE is the train of solitons leading to a comb generation. These solitons are known as dissipative Kerr soliton combs [231], see Figure 14a. The pulse dynamics is highly dependent on dispersion engineering. Attaining an anomalous GVD regime is more desirable, as the comb initialization through modulation instability at the normal GVD regime is difficult.

Nonlinear losses induced by multiphoton absorption effects such as 2PA, 3PA, and 4PA, as well as by FCA, can compromise parametric oscillations in ring resonators [232]. Concerning this issue, a number of studies reporting a numerical analysis in silicon ring resonators with the nonlinear losses taken into account have been reported [233,234]. The recombination rate of GaAs is at least three orders of magnitude larger compared to that in silicon ($10^{9}$ s${}^{-1}$ compared to $10^{3}$–$10^{6}$ s${}^{-1}$, depending on silicon’s purity) [235]. This indicates a significantly shorter free-carrier life-time in GaAs compared to that in silicon, which reduces the adverse impact of FCA on the FCG in (Al)GaAs. Therefore, frequency combs in (Al)GaAs are expected to have broader spectra and higher efficiencies with reduced restrictions on the pump power [232] (note that the rate of free-carrier generation is affected by the intensity, and a lower pump intensity can further reduce the impact of FCA).

AlGaAs-OI satisfies all the requirements for efficient FCG, including low loss, high nonlinear coefficient, and the ability for dispersion engineering. M. Pu et al. demonstrated Kerr frequency comb generation in an AlGaAs-OI MRR with the Q-factor on the order of $10^{5}$ [8], see Figure 14. The anomalous GVD and high Q-factor enabled a threshold power reduction down to <10 mW.

More recently, L. Chang et al. demonstrated the generation of a Kerr frequency microcomb in an AlGaAs-OI MRR with a very high Q-factor on the order of $10^{6}$, achieved by a reduction in the sidewall and surface roughness [196]. The device was engineered to achieve an anomalous GVD. Thanks to the very high Q-factor, the authors were able to reduce the threshold power down to 36 μW. We find it important to compare the performance of AlGaAs microring resonators with that of crystalline whispering gallery mode resonators made of CaF${}_{2}$ and MgF${}_{2}$. The latter resonators exhibit higher Q-factors ($10^{9}$) [236,237]. However, achieving the anomalous dispersion requirement in crystalline resonators is challenging because one has to apply polishing to obtain the necessary device dimensions [238]. Furthermore, AlGaAs microring resonators offer superior compactness and simplicity of integration with other on-chip devices [239].

## 7. Applications

### 7.1. Wavelength Conversion

Since the first demonstrations of the nonlinear optical effects in AlGaAs waveguides, conducted in the 1970s [14,15,16], these nonlinear optical devices have been considered for an application in wavelength conversion as compact sources capable of generating light with the frequencies outside of the range of the conventional laser sources. Specifically, it was speculated that the possibility of integrating light sources with passive wavelength conversion devices on the same platform could expand the wavelength range of the former to the frequency windows where conventional laser sources are not easily accessible.

Due to its wide transparency window (0.9–17 μm) and very high associated second-order nonlinear coefficients, AlGaAs has been envisioned as a wavelength conversion platform for various wavelength ranges. Specifically, the early wavelength conversion experiments exploit CO${}_{2}$ laser sources operating around 10 μm to achieve SHG in AlGaAs with the mid-IR output (around 5 μm [14,240,241,242]). Furthermore, the experiments on generation of mid-IR radiation around 4 μm [42] and 5 μm [47] in AlGaAs waveguides by DFG with the pump and signal sources operating in the wavelength ranges around 1 μm and 1.3 μm, respectively, have also been reported. These works pave the way toward creating fully integrated diode-pumped optical parametric oscillators in the 4–6 μm region. SHG of the fundamental 4-μm radiation, leading to the generation of 2-μm radiation in AlGaAs waveguides, has also been demonstrated [104]. Such devices, in combination with recently realized mid-IR Mach-Zender modulators [243] and microring resonators [244], can contribute to the development of fully functional AlGaAs mid-IR photonic circuits for sensing and communications.

There is a plethora of experimental works demonstrating SHG [30,38,41,43,44,45,46,100,105,106,107,108,109,110,111,112,113,114,115], SFG [48,123,124], and DFG [38,124,125,126,193] with the pump, signal, or generated output in the wavelength range around 1550 nm, below half-the-bandgap (see Table 4 for efficiency comparison). It is, however, often the case in these experiments that either SH or the pump/signal wavelength falls into the 700-nm range, where the propagation loss coefficient due to the residual Urbach’s tail absorption and scattering off the structural and epitaxial imperfections is considerable (20–150 dB/cm). Nevertheless, relatively high conversion efficiencies were demonstrated in some works [124]. In a more extreme case, despite the high absorption losses in the wavelength range above the bandgap, generation of blue and green light in AlGaAs waveguides (see the surface-emitted SHG experiments [37]) has been reported. For example, one particular experiment on the generation of visible light by SFG features the wide-band operation of on-chip wavelength converters spanning from green to red [17]. A remarkable tunability of nonlinear optical effects achieved in some works reveals the potential for integrated AlGaAs nonlinear optical devices to serve as widely tunable integrated sources in the visible [17], near-IR [124,126], and mid-IR [47] frequency ranges.

### 7.2. Optical Communication Networks

Another application of AlGaAs nonlinear integrated devices that has been explored since the 1990s is optical communication networks. In particular, all-optical signal processing and all-optical switching have been the techniques under development in AlGaAs integrated platforms since the early days of wavelength-division multiplexing (WDM) networks. The first demonstrations of optical switching were conducted in a variety of AlGaAs nonlinear optical devices operating just below half-the-bandgap [28]. Optical pulse switching was demonstrated in nonlinear direction couplers [22,24], X-switches [25], polarization switches [26], and asymmetric Mach–Zender interferometers [21]. Furthermore, efficient time–domain multiplexing [31] and demultiplexing [33,34] of optical pulses by virtue of the third-order optical nonlinearity below half-the-bandgap have been demonstrated. Moreover, optical spatial soliton propagation and interaction have been experimentally achieved in AlGaAs heterostructures with 1D confinement [39,49]. It has been shown that the unity ratio of the XPM to SPM coefficients, necessary for the spatial soliton formation, is achievable in AlGaAs [32].

All-optical logic gates are key optical signal processing components. Intensity-dependent all-optical logic gates based on critically coupled GaAs ring resonator have been demonstrated [145]. The operation of these devices is based on the change in the refractive index of GaAs induced by free carriers generated by 2PA. The benefits of using microring resonator for this purpose resided in its compact size, reduced switching threshold, and higher switching contrast.

**Figure 15:**
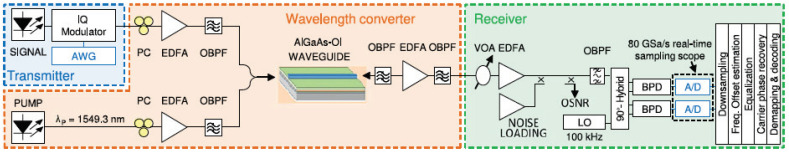
Schematic of the experimental setup of 256-QAM transmitter, AlGaAs-OI wavelength converter, and receiver. ©2017 IEEE. Reprinted, with permission, from [139].

The most recent achievements in all-optical signal processing facilitated by nonlinear optical interactions in AlGaAs waveguides include various demonstrations of data channel wavelength conversion [136,141,158,197,226]; some of them featuring simultaneous operation on multiple wavelength channels [136,156]. One example of such an achievement is reported in [139], where the authors demonstrated wavelength conversion of advanced modulation formats such as 10-GBd, 64-state quadrature-amplitude modulation (64-QAM), and 256-QAM via FWM with high conversion efficiency over 29-nm wavelength window covering almost the entire telecom C-band. In Figure 15, we show an experimental setup of such a wavelength conversion scheme. Advanced modulation formats represent the path towards improved wavelength channel utilization, where numerous degrees of freedom associated with light, such as its amplitude, phase, and polarization, are explored. All-optical wavelength conversion by virtue of nonlinear optical effects such as FWM have the benefit of operating simultaneously on several wavelength channels, coherence preservation, modulation-format transparency, and elimination of optical-to-electrical and back-to-optical conversions.

Another remarkable example features AlGaAs-OI waveguides with the dimensions optimized for higher-order dispersion and phase-matching [140]. The demonstration of ultra-broadband FWM spanning the range between 1280 and 2020 nm continuously holds promise for new-generation fiber-optics systems exploring the range around 2 μm for increased-capacity networks. Furthermore, the demonstration of ultra-high-rate wavelength conversion by FWM in such a device has been performed for a 1.28-Tbit/s return-to-zero (RZ) differential phase-shift-keyed (DPSK) optical signal.

**Figure 16:**
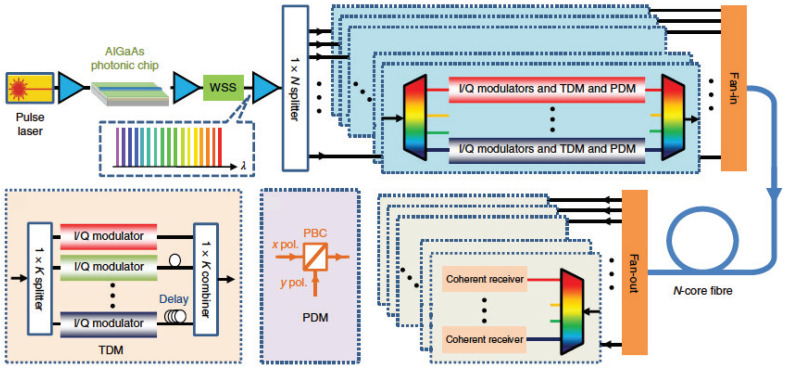
Generation and transmission of multi-100 Tbit/s data carried by an AlGaAs-OI SPM-based frequency comb. Reprinted by permission from Nature [156], ©2018.

Recent advancements in frequency-comb generation by nonlinear interactions offer the possibility of replacing multiple laser sources in parallel optical communication lines utilizing space division multiplexing by a single frequency-comb source [156]. In that study, the authors produced a stable broadband frequency comb using a narrowband frequency comb as a seed and SPM in a highly nonlinear dispersion-engineered AlGaAs-OI waveguide (see Figure 16). The remarkable achievement of this work was the very high pump-to-comb conversion efficiency of 66%, and the demonstration of the extremely high capacity of such a system. Furthermore, the authors explored all six dimensions of optical signal transmission, including the amplitude, phase, time, frequency, polarization, and space-division multiplexing, to demonstrate the transmission of 661 Tbit/s carried on the broadened frequency comb produced in a single nonlinear waveguide. Moreover, such a waveguide-based frequency-comb source operated without a temperature controller and stabilizing feedback loop, outperforming its ancestors, single-line WDM network lasers. All these results clearly demonstrate the feasibility of all-optical signal processing in AlGaAs platforms, and their mature standing for the near-future commercialization.

### 7.3. Integrated Quantum Photonics

Quantum communication, computing, and metrology exploit non-classical states of light to achieve unprecedented communication security, computation efficiency, and high measurement precision. Quantum optical setups based on bulk optical components, including single-photon sources, single-photon detectors, and linear optical circuits, have severe circuit stability, complexity and scalability limitations. Therefore, the emergence of integrated quantum photonics revolutionized the field of quantum photonic technologies, representing a robust and scalable solution for future quantum applications. A robust integrated quantum platform should exhibit the capability of monolithic integration of a source of quantum light (single-photon and bi-photon sources), single-photon detectors and photon counters, and quantum light steering and manipulation circuits. AlGaAs/GaAs integrated optics platform has been proved to represent a feasible solution capable of accommodating all these functions [245,246]. Among recent achievements in AlGaAs quantum circuit components and integration, the most notable are the developments in the field of single-photon [247] and photon-pair sources [129,130,152,248,249,250,250], single-photon detectors [251,252,253,254], and quantum switching and manipulation devices [245,246]. Moreover, attempts have been made to integrate multiple functionalities on AlGaAs/GaAs platform in a monolithic manner [245,246,253].

**Figure 17:**
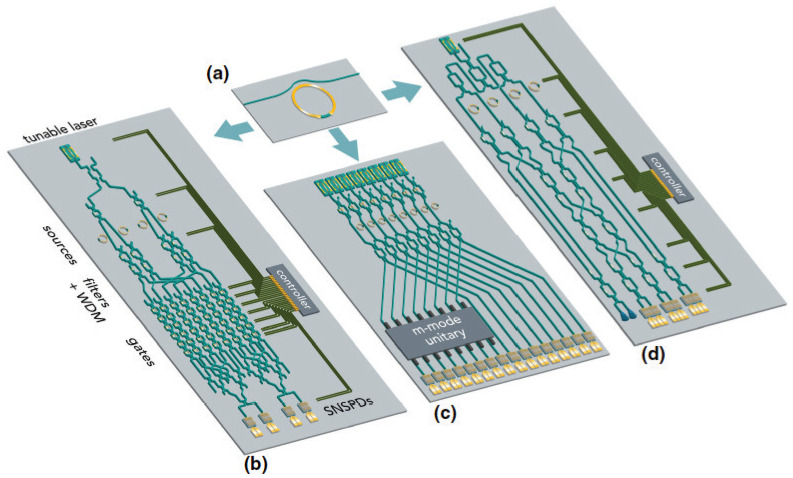
(**a**) Tunable AlGaAs-OI MRR entangled-photon-pair source. AlGaAs-OI represents a practical host platform for a monolithic integration of the source with (**b**) quantum gates for optical computers, (**c**) *m*-mode unitary operations for boson sampling, and (**d**) Bell-state measurements for chip-to-chip quantum-state teleportation. The figure is reprinted from [152].

Nonlinear optical interactions play an important role in developing integrated quantum photonic circuits based on AlGaAs. In particular, integrated sources of correlated photons relying on either second- or third-order nonlinear optical interactions have been deemed best performing and most widely used sources [247]. Photon pairs can be generated either through a $\chi^{\left(2\right)}$ effect of SPDC [129,130,131,248] (a reverse to SHG interaction) or by a $\chi^{\left(3\right)}$-based SFWM [151,152]. Both approaches have been shown to represent a viable solution for generating non-classical states of light. In Figure 17, we show an example of the monolithic integration of an AlGaAs-OI SFWM MRR integrated source of correlated photons with various on-chip quantum circuits [152].

The operation of nonlinear switching devices necessary for the manipulation and processing of quantum states of light relies on nonlinear optics. Passive nonlinear devices have been successfully implemented for single-photon switching and on-chip manipulation of quantum light [245,246].

**Figure 18:**
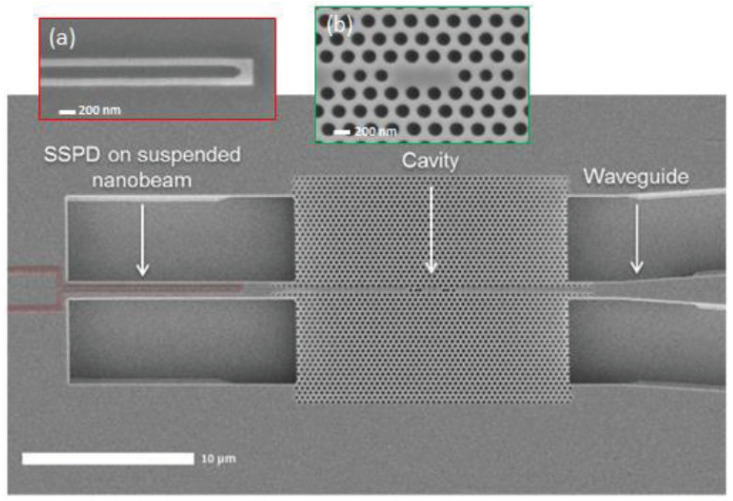
SEM image of a fully integrated single-photon source and detector. (**a**) Superconducting single-photon detector (SSPD), (**b**) photonic crystal cavity. The figure is reprinted with permissions from [253].

On the detection side, one of the technologies in development for single-photon detectors and photon counters is based on NbN supercoducting thin ribbons deposited on top of an AlGaAs waveguide, either suspended [253] or ridge [251,252]. In Figure 18, we show an example of a single-photon quantum source and detector fully integrated with AlGaAs technology [253]. While the technology has shortcomings associated with the difficulty of its technical implementation, it represents an important stepping-stone towards the realization of fully integrated turn-key quantum circuits. In fact, such integration has already been successfully implemented in proof-of-principle studies [245,246,253], which confirms the suitability of AlGaAs integrated platform for practical integrated quantum photonic circuits.

### 7.4. Challenges and Future Prospective

We reviewed the applications exploring classical nonlinear integrated devices based on AlGaAs/GaAs material platforms, such as wavelength converters and all-optical signal processing devices. These developments share the common feature of reliance on AlGaAs as a platform for monolithic integration of light sources, nonlinear passive devices, and detection schemes. The main hurdle towards realizing this ultimate goal, fully integrated circuits implementing nonlinear optical interactions, is the compatibility issue of AlGaAs nonlinear waveguide structures with the active devices platforms. Several AlGaAs nonlinear waveguide platforms approach this requirement [108,144], but none of them is easily compatible with laser fabrication requiring doped semiconductor layers. Vertical integration (for example, of the kind implemented in InP [180,181], which is similar in principle to GaAs technology) could represent one possible solution to the compatibility issue.

Implementing integrated quantum circuits with monolithically integrated single-photon sources, photon manipulation circuitry, and single-photon detectors appears to be even more challenging. Specifically, the challenge resides in the fabrication technologies compatibility of the three crucial circuit parts and the manipulation of the detectors typically requiring cryogenic temperatures [246,253]. Mitigation of technological incompatibilities remains an open issue to be addressed by future research.

Additional developments are required to improve the performance of integrated quantum optical circuits. Specifically, the passive building blocks such as beam splitters and couplers would benefit from further optimization to reduce the coupling losses for better scalability towards multiple-cubit circuits [246]. Furthermore, certain integrated optical components are still missing or lack optimal designs. Specifically, integrated photonic filters and single-photon buffers preserving non-classical properties of light are necessary for photon manipulation [246]. Classical and quantum optical memory represents a crucial part of functional integrated optical circuits while still remaining in the infancy of its development. Propagation loss reduction enabled by an invent of new low-loss high-index contrast AlGaAs waveguide platforms such as AlGaAs-OI and suspended air-cladded waveguides, is expected to address another pressing need for efficient and broadband all-optical signal processing devices with ultra-low energy consumption.

Another challenge that concerns up-scaling of integrated quantum circuits to meet the practical requirements for their complexity resides in the limited refractive index contrast in AlGaAs heterostructures. Furthermore, single-photon emission and manipulation is especially sensitive to even minor propagation losses. Both these issues are being successfully addressed by the implementation of (Al)GaAs-OI with superior refractive-index contrast compared to that of the heterostructures and record-low propagation losses [12] that can allow more components to be accommodated for applications requiring system-level integration.

Nevertheless, remarkable progress in addressing these challenges has been demonstrated. The proof-of-principle AlGaAs photonic circuits with nonlinear optical functionalities, as described in Section 7.1, Section 7.2 and Section 7.3, indicate that the technological compatibility challenges are being addressed. Continuous improvements in wavelength-conversion efficiencies [9,10,11,140], quantum-source brightness [152], and on-chip detection techniques [246,253] already make integrated quantum photonic circuits and nonlinear devices more appealing for large-scale implementation compared to their free-space counterparts [246]. As such, AlGaAs holds promise for practical implementation in optical communication networks, nonlinear integrated sources covering a wide spectral range, and scalable integrated quantum photonic circuits.

## 8. Conclusions

This manuscript provided an overview of the existing experimental studies of the nonlinear optical performances of AlGaAs waveguide platforms and geometries. Furthermore, we discussed the existing approaches toward realizing highly efficient nonlinear integrated devices.

Thanks to the large values of the refractive index and $\chi^{\left(2\right)}$ and $\chi^{\left(3\right)}$ nonlinear optical coefficients, AlGaAs integration platforms provide tight light confinement and efficient nonlinear optical interactions. Moreover, AlGaAs is naturally suited for a monolithic integration of both passive and active devices on the same chip. Consequently, AlGaAs has excellent potential to be implemented in a wide range of applications, such as all-optical signal processing, spectroscopy, high-efficiency light sources, and integrated quantum circuits.

We considered several AlGaAs waveguide platforms that can be differentiated based on the number of layers and type of claddings. To date, AlGaAs-OI represents the best solution for highly efficient nonlinear photonic devices, thanks to its very low propagation loss and a high potential for dispersion engineering. These features point to a bright future for AlGaAs monolithic circuits with nonlinear functionalities.

## Figures and Tables

**Table 1:** Nonlinear waveguide materials and their optical coefficients at 1550 nm unless stated otherwise.

Material	$n_{0}$	χ${}^{\left(2\right)}$ (pm/V)	$n_{2}$ ($10^{-14}\;{\mathrm{cm}}^{2}/W$)	α${}_{2}$ (cm/GW)	$L_{\min}$ (dB/cm)
GaAs *	3.4	238 at 1533 nm [103]	20 [157]	15 [158]	0.4 [12]
${\mathrm{Al}}_{0.18}{\mathrm{Ga}}_{0.82}\mathrm{As}$ *	3.28	226 at 1533 nm ^⊛^ [102,103]	15 [13]	0.05 [9]	0.56 [9]
${\mathrm{In}}_{0.63}{\mathrm{Ga}}_{0.37}{\mathrm{As}}_{0.8}P_{0.2}$ *	3.58	^†^ [159,160]	10 [86]	19 [86]	3 [86]
GaP *	3.05	163 at 1318 nm [161]	6 [93]	–	1.2 [93]
${\mathrm{In}}_{0.49}{\mathrm{Ga}}_{0.51}P$ *	3.1	220 at 1579 nm [162]	4 [163]	–	10 [96]
GaN *	2.3	−9.2 at 1064 nm [164]	1.4 [165]	–	0.17 [165]
AlN *	2.12	8.6 at 1030 nm [166]	0.23 [89]	–	0.42 [167]
Si	3.47	40 at 2313 nm ^‡^ [168]	4.5 [169]	0.79 [169]	0.18 [170]
${\mathrm{As}}_{2}S_{3}$	2.38	–	2.92 [78]	$6.2\times 10^{-4}$ [79]	0.05 [78]
${\mathrm{Si}}_{3}N_{4}$	2.0	–	0.25 [171]	–	0.06 [65]
${\mathrm{LiNbO}}_{3}$ *	2.21	−41.2 at 1.52μm [74]	0.18 [172]	–	2.5 [173]
Hydex [85]	1.7	–	0.12	–	0.06
${\mathrm{SiO}}_{2}$ [174]	1.4	–	0.022	–	N/A

* Materials exhibiting $\chi^{\left(2\right)}$ interactions. The values of the largest $\chi^{\left(2\right)}$ tensor components are specified. ^⊛^ Calculated from the relation *d*(Al_0.18_Ga_0.82_As) = 0.95*d*(GaAs) [102], with *d*(GaAs) = 119 pm/V at 1533 nm [103]. ^†^ The value of $\chi^{\left(2\right)}$ is unavailable in literature, however some sources [159,160] claim that it is “at least four times larger than that of GaAs”. ^‡^ Silicon does not exhibit intrinsic $\chi^{\left(2\right)}$. However, it was detected and measured in strained silicon [168,175] (the table shows the corresponding value for SHG).

**Table 2:** Nonlinear coefficients of AlGaAs waveguides.

Guiding Layer	Platform	Geometry ^a^	Wavelength (nm)	γ ${(m}^{-1}W^{-1})$	Mode	Technique ^b^	Refs.
${\mathrm{Al}}_{0.17}{\mathrm{Ga}}_{0.82}\mathrm{As}$ ^c^	AlGaAs-OI	NW	1538, 1544	720	TE	FWM	[7]
	AlGaAs-OI	NW	1549, 1550	630	TE	FWM	[140]
${\mathrm{Al}}_{0.18}{\mathrm{Ga}}_{0.82}\mathrm{As}$	three-layer AlGaAs	NW	1530, 1550	180	TE	FWM	[144]
	three-layer AlGaAs	HC	1530, 1550	90	TE	FWM	[144]
	three-layer AlGaAs	SL	1540, 1560	51	TE	FWM	[144]
${\mathrm{Al}}_{0.21}{\mathrm{Ga}}_{0.79}\mathrm{As}$ ^d^	AlGaAs-OI	NW	1545, 1550	350	TE	FWM	[141]
${\mathrm{Al}}_{0.24}{\mathrm{Ga}}_{0.76}\mathrm{As}$	three-layer AlGaAs	SL	1530	5.9	TE	SPM	[52]
${\mathrm{Al}}_{0.25}{\mathrm{Ga}}_{0.75}\mathrm{As}$	three-layer AlGaAs	NW	1554.9, 1555	150	-	FWM	[138,151]

^a^ Waveguide geometries: SL—strip-loaded, HC—half-core, and NW—nanowire. ^b^ SPM—self-phase modulation and FWM—four-wave mixing. ^c^ Composition given in Ref. [8]. ^d^ Composition given in Ref. [139].

**Table 3:** Second-order nonlinear coefficient.

Material ^a^	Platform (geom.) ^b^	Wavelength ^c^ (nm)	*d* (pm/V)	Additional Information	Technique ^d^	Ref.
GaAs	GaAs-OI (RD)	1968	180	TE mode	SHG	[10]
GaAs	Bulk	500–1340	375 (852nm)	$d=\chi_{xyz}^{\left(2\right)}/2$	SHG	[204]
	Bulk	1064	170	$d_{36}$	SHG	[103]
	Bulk	1533	119	$d_{36}$	SHG	[103]
	Bulk	4135	94	$d_{14}$	SHG	[104]
	Bulk	10,600	83	$d_{36}$	SHG	[205]
${\mathrm{Al}}_{0.61}{\mathrm{Ga}}_{0.39}\mathrm{As}$	BRW (SL)	1555	40	TM (pump) TE (signal)	SFG	[123]
${\mathrm{Al}}_{0.2}{\mathrm{Ga}}_{0.8}\mathrm{As}$	Thin-film	1064	158 ^e^	$d=0.93\;d_{\mathrm{GaAs}}$	SHG	[102]
${\mathrm{Al}}_{0.4}{\mathrm{Ga}}_{0.6}\mathrm{As}$	Thin-film	1064	129 ^e^	$d=0.76\;d_{\mathrm{GaAs}}$	SHG	[102]
${\mathrm{Al}}_{0.6}{\mathrm{Ga}}_{0.4}\mathrm{As}$	Thin-film	1064	97 ^e^	$d=0.57\;d_{\mathrm{GaAs}}$	SHG	[102]
${\mathrm{Al}}_{0.8}{\mathrm{Ga}}_{0.2}\mathrm{As}$	Thin-film	1064	61 ^e^	$d=0.36\;d_{\mathrm{GaAs}}$	SHG	[102]
AlAs	Thin-film	1064	39 ^e^	$d=0.23\;d_{\mathrm{GaAs}}$	SHG	[102]

^a^ For waveguides, this column reports the guiding layer’s material composition. ^b^ Waveguide geometries: RD—ridge, SL—strip-loaded, BRW—Bragg reflector waveguide, and GaAs-OI—GaAs-on-insulator platform. ^c^ The wavelength range is reported only for the works where the spectral dependence of the nonlinear coefficient was measured. In these cases, the table reports the maximum nonlinear coefficient in the range. ^d^ SHG—secondharmonic generation and SFG—sum-frequency generation. ^e^ *d*_AlGaAs_ was calculated using *d*_GaAs_ = 170 pm/V from Ref. [103].

**Table 4:** Normalized conversion efficiencies (CE) and phase-matching mechanisms for second-order processes in AlGaAs waveguides.

Guiding Layer	Process	Platf. ^a^	Geom. ^b^	Phase-Match. Mechanism ^c^	λ ${}_{\mathrm{inc}}$ (nm)	λ ${}_{\mathrm{genr}}$ (nm)	CE ^d^ [%/(W cm${}^{2}$)]	Ref.
${\mathrm{Al}}_{0.25}{\mathrm{Ga}}_{0.75}\mathrm{As}/{\mathrm{AlO}}_{x}$	SFG	ox-AlGaAs	RD	BPM	1543 (CW) 1550 (CW)	773	1080	[124]
${\mathrm{Al}}_{0.6}{\mathrm{Ga}}_{0.4}\mathrm{As}$	SFG	AlGaAs	SL	PDI-QPM	1540 (CW) 1575 (CW)	779	810	[48]
${\mathrm{Al}}_{0.61}{\mathrm{Ga}}_{0.395}\mathrm{As}$	SFG	BRW	SL	MPM	1555 (CW) 1552 (CW)	777	298	[123]
${\mathrm{Al}}_{0.25}{\mathrm{Ga}}_{0.75}\mathrm{As}/{\mathrm{AlO}}_{x}$	DFG	ox-AlGaAs	RD	BPM	773 (CW) 1559 (CW)	1533	9.7 ^e^	[124]
$\mathrm{GaAs}/{\mathrm{Al}}_{0.85}{\mathrm{Ga}}_{0.15}\mathrm{As}$	DFG	SLWG	SL	PDD-QPM	793 (CW) 1535–1555 (CW)	1618–1640	0.072	[126]
${\mathrm{Al}}_{0.61}{\mathrm{Ga}}_{0.39}\mathrm{As}$	DFG	BRW	SL	MPM	778 (CW) 1546 (CW)	1566	0.058 ^e^	[125]
GaAs	SHG	GaAs-OI	RD	MPM	2000 (CW)	1000	47,000	[10]
${\mathrm{Al}}_{0.19}{\mathrm{Ga}}_{0.81}\mathrm{As}$	SHG	Suspended	NW	BPM	1594 (CW)	797	12,800	[121]
${\mathrm{Al}}_{0.61}{\mathrm{Ga}}_{0.39}\mathrm{As}$	SHG	BRW	SL	MPM	1556 (1.8ps)	778	11,400	[111]
${\mathrm{Al}}_{0.27}{\mathrm{Ga}}_{0.73}\mathrm{As}$	SHG	AlGaAs-OI	NW	MPM	1560 (1.8ps)	780	1202	[11]
${\mathrm{Al}}_{0.25}{\mathrm{Ga}}_{0.75}\mathrm{As}/{\mathrm{AlO}}_{x}$	SHG	ox-AlGaAs	RD	BPM	1544 (CW)	772	1120	[100]
${\mathrm{Al}}_{0.6}{\mathrm{Ga}}_{0.4}\mathrm{As}$	SHG	AlGaAs	SL	PDI-QPM	1557 (CW)	779	1040	[45]
$\mathrm{GaAs}/{\mathrm{Al}}_{0.85}{\mathrm{Ga}}_{0.15}\mathrm{As}$	SHG	SLWG	SL	PDD-QPM	1576 (1.9ps)	788	350	[113]
${\mathrm{Al}}_{0.67}{\mathrm{Ga}}_{0.33}\mathrm{As}$	SHG	AlGaAs	RD	OP-QPM	1563 (CW)	782	67.2	[210]
${\mathrm{Al}}_{0.2}{\mathrm{Ga}}_{0.8}\mathrm{As}$	SHG	AlGaAs	NW	MPM	1582 (CW)	791	13.8	[115]

^a^ Multilayer platforms: ox-AlGaAs—alternating AlO*_x_* and Al*_x_*Ga*_1−x_*As layers, SLWG—superlattice waveguide, and BRW—Bragg reflector waveguide. AlGaAs represents the two- or three-layer platforms with different aluminum content in each layer, while (Al)GaAs-OI represents the (Al)GaAs-on-insulator. ^b^ Geometries: RD—ridge, SL—strip-loaded, and NW—nanowire. ^c^ BPM—birefrincence phase-matching, PDI-QPM—periodic domain inversion quasi-phase matching, MPM—modal phase-matching, PDD-QPM—periodic domain disordering quasiphase matching, and OP-QPM—orientation-patterned quasi-phase matching. ^d^ For SHG: CE = [*P*_SH_/($P_{p}^{2}$*L*^2^)] × 100%. For SFG and DFG: CE = [*P*_i_/(*P*_p_*P*_s_*L*^2^)] × 100%. *L* is the waveguide length, while *P*_SH_, *P*_p_, *P*_i_, and *P*_s_ are the second-harmonic, pump, idler, and signal powers, respectively. ^e^ external powers were used to calculate the efficiency.

**Table 5:** Nonlinear refractive indices measured in (Al)GaAs waveguides.

Material ^a^	Platform ^b^	Geom. ^c^	Wavelength ^d^ (nm)	$n_{2}$ ${(10}^{-13}\;{\mathrm{cm}}^{2}/W)$	Additional Information	Technique ^e^	Refs.
GaAs	Bulk		1680–3250	3.0 (1680nm)	Along [110]	Z-scan	[157]
GaAs/${\mathrm{Al}}_{0.2}{\mathrm{Ga}}_{0.8}\mathrm{As}$	MQW	SL	1545	1.3		NLDC	[22]
GaAs/${\mathrm{Al}}_{0.3}{\mathrm{Ga}}_{0.7}\mathrm{As}$	MQW	HC	1620	0.87	TE mode	SPM	[19]
	MQW	HC	1620	0.54	TM mode	SPM	[19]
GaAs/${\mathrm{Al}}_{0.4}{\mathrm{Ga}}_{0.6}\mathrm{As}$	MQW	SL	1550	2.6	TE mode	SPM	[40]
	MQW	SL	1550	3.3	TM mode	SPM	[40]
GaAs/${\mathrm{Al}}_{0.85}{\mathrm{Ga}}_{0.15}\mathrm{As}$	SLWG	SL	1525–1605	2.8 (1545nm)	TE mode	SPM	[150]
	SLWG	SL	1525–1605	1.1 (1525nm)	TM mode	SPM	[150]
GaAs/AlAs	SLWG	SL	1505–1625	5.5 (1505nm)	TE mode	SPM	[146]
	SLWG	SL	1505–1625	2.4 (1505nm)	TM mode	SPM	[146]
${\mathrm{Al}}_{0.14}{\mathrm{Ga}}_{0.86}\mathrm{As}$ ^f^	AlGaAs	NW	1552.45, 1551.9	2.3	TE mode	FWM	[135]
${\mathrm{Al}}_{0.17}{\mathrm{Ga}}_{0.83}\mathrm{As}$ ^f^	AlGaAs	NW	1552.45, 1551.9	1.5	TE mode	FWM	[135]
${\mathrm{Al}}_{0.24}{\mathrm{Ga}}_{0.76}\mathrm{As}$ ^f^	AlGaAs	NW	1552.45, 1551.9	0.9	TE mode	FWM	[135]
${\mathrm{Al}}_{0.26}{\mathrm{Ga}}_{0.74}\mathrm{As}$ ^f^	AlGaAs	NW	1552.45, 1551.9	0.85	TE mode	FWM	[135]
${\mathrm{Al}}_{0.18}{\mathrm{Ga}}_{0.82}\mathrm{As}$	AlGaAs	SL	1545	1.1	See Figure 10a	NLDC	[22]
	AlGaAs	SL	1490–1660	2.4 (1500nm)	See Figure 10a	SPM	[13,23,36,39]
	AlGaAs	NW	1552.45, 1551.9	1.1	See Figure 10a	FWM	[9]
${\mathrm{Al}}_{0.2}{\mathrm{Ga}}_{0.8}\mathrm{As}$	AlGaAs	SL	1620	0.36		SPM	[19]
${\mathrm{Al}}_{0.23}{\mathrm{Ga}}_{0.77}\mathrm{As}$	AlGaAs	RD	1600	0.21	TM (pump) TE (signal)	MZI	[35]

^a^ For waveguides, this column reports the guiding layer’s composition. ^b^ Multi-layer platforms: MQW—multi-quantum-well waveguide and SLWG—superlattice waveguide. AlGaAs represents the two- or three-layer platforms with different aluminum content in each layer. ^c^ Waveguide geometries: SL—strip-loaded, HC—halfcore, NW—nanowire, and RD—ridge. ^d^ The wavelength range is reported only for the works in which the spectral dependence of the nonlinear coefficient was measured. In these cases, the table reports the maximum nonlinear coefficient in the range. ^e^ NLDC—nonlinear directional coupler switching, SPM—self-phase modulation, FWM—four-wave mixing, and MZI—Mach-Zehnder interferometry. ^f^ Composition calculated from the bandgap energy [99].

**Table 6:** Nonlinear absorption coefficients.

Material ^a^	Platform ^b^	Geom. ^c^	Wavelength ^d^ (nm)	Nonlinear Coefficient	Additional Information	Technique ^e^	Refs.
				$\alpha_{2}$ (cm/GW)			
GaAs	Bulk		1300–1700	20 (1300nm)	Along [110]	NLT	[157]
GaAs	AlGaAs	RD	1475–1700	33 (1475nm)		NLT	[29]
GaAs/${\mathrm{Al}}_{0.2}{\mathrm{Ga}}_{0.8}\mathrm{As}$	MQW	SL	1545	0.08		NLDC	[22]
GaAs/${\mathrm{Al}}_{0.26}{\mathrm{Ga}}_{0.74}\mathrm{As}$	MQW	SL	1064	22	TE mode	NLT	[216]
	MQW	SL	1064	15	TM mode	NLT	[216]
	MQW	SL	1064	13	TM (pump) TE (probe)	NLT	[217]
GaAs/${\mathrm{Al}}_{0.3}{\mathrm{Ga}}_{0.7}\mathrm{As}$	MQW	HC	1670	0.65	TE mode	SPM	[19]
	MQW	HC	1670	0.4	TM mode	SPM	[19]
GaAs/AlAs	SLWG	SL	1505–1625	3.9 (1505nm)	TE mode	NLT	[146]
	SLWG	SL	1505–1625	1.1 (1505nm)	TM mode	NLT	[146]
${\mathrm{Al}}_{0.14}{\mathrm{Ga}}_{0.86}\mathrm{As}$ ^f^	AlGaAs	NW	1550	3.3	TE mode	NLT	[135]
${\mathrm{Al}}_{0.18}{\mathrm{Ga}}_{0.82}\mathrm{As}$	AlGaAs	SL	1545	0.08	See Figure 10b	NLDC	[22]
	AlGaAs	SL	1480–1660	1.1 (1480nm)	See Figure 10b	NLT	[13,23,148]
	AlGaAs	NW	1552.45, 1551.9	0.05	See Figure 10b	FWM	[9]
	AlGaAs	NW	1480–1540	2.3 (1480nm)	See Figure 10b	NLT	[148]
	AlGaAs	HC	1480–1560	2.4 (1480nm)	See Figure 10b	NLT	[148]
${\mathrm{Al}}_{0.2}{\mathrm{Ga}}_{0.8}\mathrm{As}$	AlGaAs	SL	1670	0.026		NLT	[19]
${\mathrm{Al}}_{0.23}{\mathrm{Ga}}_{0.77}\mathrm{As}$	AlGaAs	RD	1600	0.06	TM (pump) TE (signal)	MZI	[35]
				$\alpha_{3}$ $\left({\mathrm{cm}}^{3}/{\mathrm{GW}}^{2}\right)$			
GaAs	Bulk		1850–2500	0.35 (2300nm)	Along [110]	NLT	[157]
GaAs/${\mathrm{Al}}_{0.2}{\mathrm{Ga}}_{0.8}\mathrm{As}$	MQW	SL	1545	0.16		NLDC	[22]
${\mathrm{Al}}_{0.17}{\mathrm{Ga}}_{0.83}\mathrm{As}$ ^f^	AlGaAs	NW	1550	0.083	TE mode	NLT	[135]
${\mathrm{Al}}_{0.18}{\mathrm{Ga}}_{0.82}\mathrm{As}$	AlGaAs	SL	1500–1660	0.13 (1660nm)	See Figure 10c	NLT	[13,22,28]
${\mathrm{Al}}_{0.2}{\mathrm{Ga}}_{0.8}\mathrm{As}$	AlGaAs	SL	1670	0.004		NLT	[19]
	AlGaAs	SL	1550	0.08	TM mode	NLT	[134]

^a^ For waveguides, this column reports the guiding layer’s composition. ^b^ Multi-layer platforms: MQW—multi quantum-well waveguide and SLWG—superlattice waveguide. AlGaAs represents the two- or three-layerplatforms with different aluminum content in each layer. ^c^ Waveguide geometries: SL—strip-loaded, HC—half-core, and NW—nanowire. ^d^ The wavelength range is reported only for the references where the spectral dependence of the nonlinear coefficient was measured. In these cases, the table reports the maximum nonlinear coefficient in the range. ^e^ NLT—Nonlinear transmittance, NLDC—nonlinear directional coupler switching, SPM—self-phase modulation, FWM—four-wave mixing, and MZI—Mach-Zehnder interferometry. ^f^ Composition calculated from the bandgap energy [99].

**Table 7:** Conversion efficiencies (CE) reported for FWM in AlGaAs waveguides (WG) and microring resonators (MRR).

Guiding Layer	Platform ^a^	Geometry ^b^	$\lambda_{\mathrm{pump}}$ (nm)	$\lambda_{\mathrm{signal}}$ (nm)	CE (dB)	BW ^c^ (nm)	Enhanc. Factor ^d^ (dB)	Ref.
${\mathrm{Al}}_{0.17}{\mathrm{Ga}}_{0.83}\mathrm{As}$ ^e^	AlGaAs-OI	NW	1550 (CW)	1549 (CW)	−4.2	750	-	[140]
${\mathrm{Al}}_{0.18}{\mathrm{Ga}}_{0.82}\mathrm{As}$	AlGaAs	NW	1520 (3ps)	1540 (CW)	−5.0	78	-	[144]
${\mathrm{Al}}_{0.18}{\mathrm{Ga}}_{0.82}\mathrm{As}$	AlGaAs	NW	1552.5 (CW)	1551.9 (CW)	−7.8	123	-	[9]
GaAs/AlGaAs	QW	RD	848 (6ps)	841 (6ps)	−8.5	20	-	[20]
${\mathrm{Al}}_{0.21}{\mathrm{Ga}}_{0.79}\mathrm{As}$	AlGaAs-OI	NW	1549.5 (CW)	1540 (CW)	−12	106	-	[139]
${\mathrm{Al}}_{0.17}{\mathrm{Ga}}_{0.83}\mathrm{As}$ ^e^	AlGaAs-OI	NW MRR	1540 (CW)	1535 (CW)	−12	-	50	[140]
${\mathrm{Al}}_{0.25}{\mathrm{Ga}}_{0.75}\mathrm{As}$	AlGaAs	NW MRR	1555 (CW)	1574 (CW)	−43	-	12	[138]
GaAs	AlGaAs	NW MRR	1549 (50ps)	1564 (CW)	−44.6	-	26	[132]

^a^ AlGaAs-OI represents the AlGaAs-on-insulator waveguide platform, while AlGaAs represents the two- or three-layer platforms with different aluminum content in each layer. QW represents AlGaAs quantum-well waveguides. ^b^ Geometries: RD—ridge waveguide, NW—nanowire waveguide, and NW MRR—nanowire microring resonator. ^c^ BW—conversion bandwidth defined as signal-to-idler wavelength separation at the point where the conversion efficiency decreases by 3 dB. ^d^ The enhancement factor is calculated by comparing the conversion efficiency in a MRR to the efficiency in a straight waveguide with the same physical length. ^e^ Composition given in Ref. [8].

## Appendix A. Theoretical

Kerr coefficient $n_{2}$ and the 2PA coefficient $\alpha_{2}$ are crucial parameters in evaluating the performance of all-optical devices. It is thus important to be able to calculate these characteristics and their wavelength and polarization dependencies. To this end, several theoretical models based on the band diagrams of the semiconductor materials that allow one to obtain such dependencies have been developed [219,220,221,222,224,225]. This appendix is aimed at familiarizing the reader with the existing formalisms for estimating the dispersion of $\alpha_{2}$ and $n_{2}$, and their polarization dependencies.

The first step in all these approaches is to calculate the band structure. In this regard, the perturbative method of k·p, where k is the wave vector and p is the momentum of an electron, is used to obtain the energy of each band $E_{n}$ as a function of the wave vector k [223]. Here, three cases can be distinguished. First, one can apply the k·p formalism in the simplest form and obtain the simplest two-parabolic-band model that gives an accurate prediction of $\alpha_{2}$ values [224]. The values of $n_{2}$ can then be deduces via Kramers–Krönig (KK) relationships. This approach has been known to describe dispersion of $\alpha_{2}$ and $n_{2}$; however, it does not provide any information about the anisotropy and polarization dependencies of these parameters. The second approach, known as Kane model, is to incorporate the spin-orbit coupling into the k·p method. This approach empowers the method of obtaining four-band structure including the spin-orbit split-off valence, light-hole, heavy-hole, and conduction bands [222]. This method is sufficient to describe the dispersion of the coefficients $\alpha_{2}$ and $n_{2}$ [221]. The third method assumes an additional conduction band (the second, higher-order conduction band) and results in vectorial detail of an anisotropic band structure. The latter method allows one to predict anisotropy and polarization dependencies of $\alpha_{2}$ and $n_{2}$ [219,220].

To explore the dispersion of $\alpha_{2}$ using the quantum formalism based on the second-order perturbation theory, the electron transition rate *M* corresponding to a four-band model is first calculated [222]. Then the 2PA coefficient is expressed through *M* using the relationship $\alpha_{2}=\left(2\hbar \omega /I\right)M$ [222,224]:

(A1) $$\alpha_{2}\left(2\omega \right)=K\frac{\sqrt{E_{p}}}{n_{0}^{2}E_{g}^{3}}F_{2}\left(\frac{2\hbar \omega }{E_{g}}\right).$$

Here *K* and $E_{p}$ denote material-independent constants, *ℏ*, $E_{g}$, and *I* denote the reduced Planck constant, bandgap energy, and the intensity of light, respectively, and $F_{2}$ represents a function of the photon energy to the bandgap energy ratio. Note that $\alpha_{2}$ and $\chi^{\left(3\right)}$ are related as α2=3ω/(2ε0n02c2)Imχ(3) [255]. As can be seen from Equation (A1), $\alpha_{2}$ scales as ${\left(n_{0}^{2}E_{g}^{3}\right)}^{-1}$.

Two-parabolic-band model and nonlinear KK relationship have been used to characterise the dispersion and sign of $n_{2}$ with a good precision [224]. The resulting expression for the dispersion of $n_{2}$ has the form

(A2) $$n_{2}\left(\mathrm{esu}\right)=K^{{}^{\prime }}\frac{G_{2}\left(\frac{\hbar \omega }{E_{g}}\right)}{n_{0}^{2}E_{g}^{4}},$$

where $K^{{}^{\prime }}=3.4\times 10^{-8}$. The term $G_{2}\left(x\right)$ is the dispersion function defined as

(A3) $$G_{2}\left(x\right)=\frac{-2+6x-3x^{2}-x^{3}-\frac{3}{4}x^{4}-\frac{3}{4}x^{5}+2{\left(1-2x\right)}^{3/2}\Theta \left(1-2x\right)}{64x^{6}},$$

where Θ is the Heaviside step function. Note that n2=3/(4ε0n02c)Reχ(3) [255]. As can be seen from Equation (A2), $n_{2}$ scales as ${\left(n_{0}^{2}E_{g}^{4}\right)}^{-1}$. As discussed earlier in this section, in order to determine the anisotropy of the third-order susceptibility through the density matrix approach, one needs to account for the higher-order conduction band, namely $\Gamma_{15}^{c}$ [219].

GaAs is a zinc-blende semiconductor with 21 non-zero third-order susceptibility tensor elements, only three of which are independent (namely, $\chi_{xxxx}^{\left(3\right)}$, $\chi_{xyxy}^{\left(3\right)}$, and $\chi_{xxyy}^{\left(3\right)}$). As a result, the third-order susceptibility tensor includes non-zero off-diagonal elements that are responsible for the anisotropy of $n_{2}$ and $\alpha_{2}$. The two-photon absorption of a zinc-blende crystal can be calculated as [219,225]

(A4) α2ω=32ωε0n02c2Imχxxxx(3)∑+2Imχxyxy(3)1−∑+Imχxxyy(3)e^.e^2−∑,

where $\sum =\sum_{r}{\left|e_{r}\right|}^{4}$. The terms $e_{r}$ and $\hat{e}$ represent direction cosines with respect to the three crystalline axes (x,y,z) and the polarization unit vector of the illuminating wave, respectively. D. C. Hutchings and B. S. Wherrett found 70% and 55% variation in $\alpha_{2}$ and $n_{2}$, respectively, as the polarization of the illuminating light changed with regard to the optical axis of the GaAs crystal [219,220].
